# The potential impact of new Andean dams on Amazon fluvial ecosystems

**DOI:** 10.1371/journal.pone.0182254

**Published:** 2017-08-23

**Authors:** Bruce R. Forsberg, John M. Melack, Thomas Dunne, Ronaldo B. Barthem, Michael Goulding, Rodrigo C. D. Paiva, Mino V. Sorribas, Urbano L. Silva, Sabine Weisser

**Affiliations:** 1 Environmental Dynamics Department, National Institute for Amazon Research, Manaus, Amazonas, Brazil; 2 Bren School of Environmental Science and Management, University of California, Santa Barbara, California, United States of America; 3 Emilio Goeldi Museum of Pará, Belém, Pará, Brazil; 4 Wildlife Conservation Society, New York, New York, United States of America; 5 Institute of Hydraulic Research, Federal University of Rio Grande do Sul, Porto Alegre, Rio Grande do Sul, Brazil; 6 National Center for Research and Conservation of Amazon Biodiversity, Chico Mendes Institute for Biodiversity Conservation, Manaus, Amazonas, Brazil; 7 Department of Biology, University of Konstanz, Konstanz, Germany; Bristol University/Remote Sensing Solutions Inc., UNITED STATES

## Abstract

Increased energy demand has led to plans for building many new dams in the western Amazon, mostly in the Andean region. Historical data and mechanistic scenarios are used to examine potential impacts above and below six of the largest dams planned for the region, including reductions in downstream sediment and nutrient supplies, changes in downstream flood pulse, changes in upstream and downstream fish yields, reservoir siltation, greenhouse gas emissions and mercury contamination. Together, these six dams are predicted to reduce the supply of sediments, phosphorus and nitrogen from the Andean region by 69, 67 and 57% and to the entire Amazon basin by 64, 51 and 23%, respectively. These large reductions in sediment and nutrient supplies will have major impacts on channel geomorphology, floodplain fertility and aquatic productivity. These effects will be greatest near the dams and extend to the lowland floodplains. Attenuation of the downstream flood pulse is expected to alter the survival, phenology and growth of floodplain vegetation and reduce fish yields below the dams. Reservoir filling times due to siltation are predicted to vary from 106–6240 years, affecting the storage performance of some dams. Total CO_2_ equivalent carbon emission from 4 Andean dams was expected to average 10 Tg y^-1^ during the first 30 years of operation, resulting in a MegaWatt weighted Carbon Emission Factor of 0.139 tons C MWhr^-1^. Mercury contamination in fish and local human populations is expected to increase both above and below the dams creating significant health risks. Reservoir fish yields will compensate some downstream losses, but increased mercury contamination could offset these benefits.

## Introduction

Increased demand for electric energy in South America has led to ambitious plans for building as many as 277 new hydroelectric dams in the Amazon River Basin [1], one of the last major river systems that is largely unregulated. These plans include the construction of as many as 151 dams with capacities larger than 2 MegaWatts (MW) in the western Amazon during the next two decades [2]. Most of these dams would be built in the Andes Mountain where steep topography facilitates the creation of deep storage reservoirs with high hydraulic head. Six dams, planned for construction on major Andean tributaries with high suspended sediment concentrations, are of particular concern because they will be the largest and farthest downstream storage dams on their respective tributaries [2]. Together, these impoundments could have major impacts on the hydrology, geomorphology, biogeochemistry, biodiversity and productivity of the Amazon River system, affecting human livelihoods and well-being from the headwaters to the estuary. Significant changes are expected both downstream and upstream of these dams.

**Fig 1:**
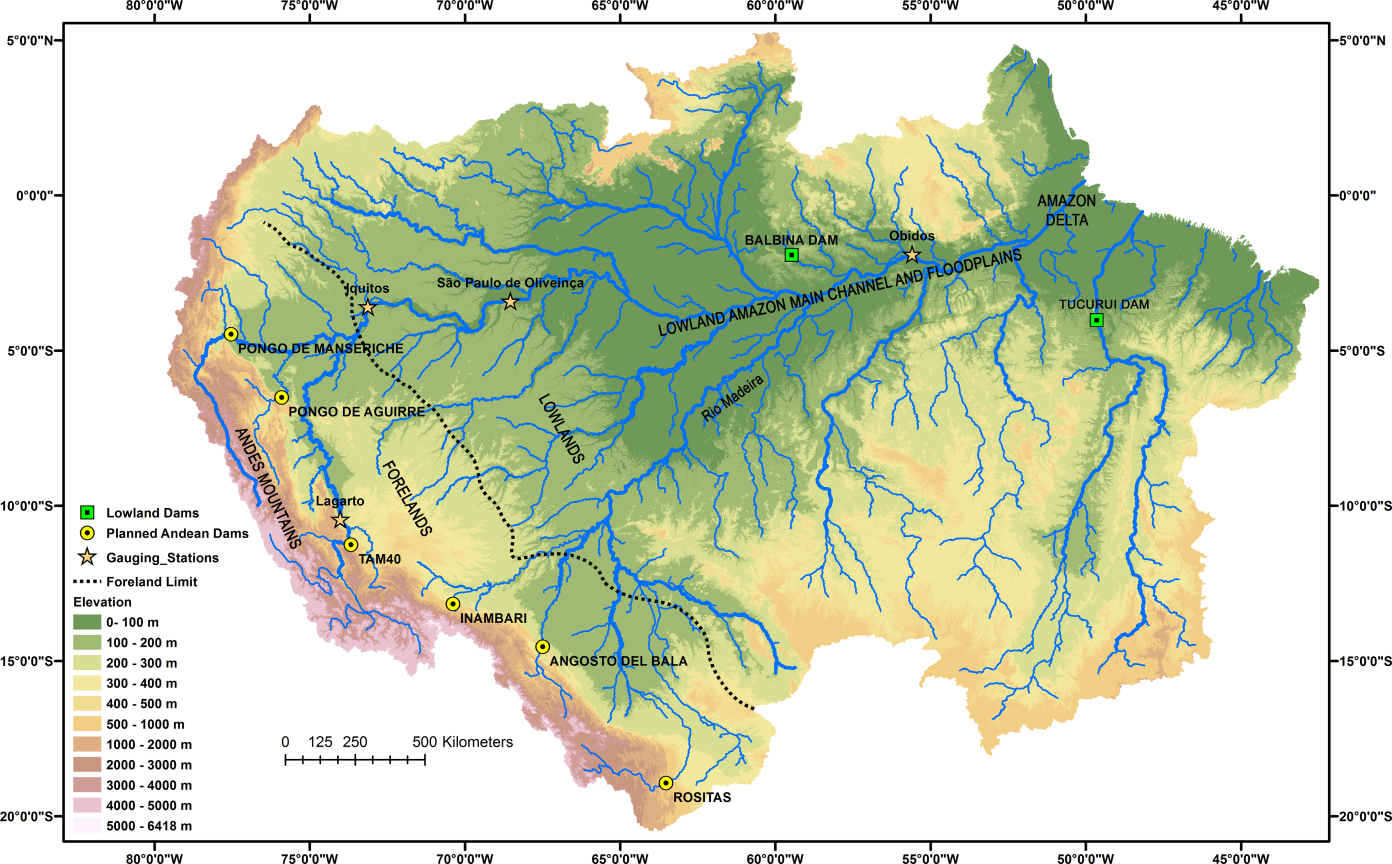
Amazon basin, showing main hydrological and geomorphological features and locations of proposed Andean dams. Locations of river gauging stations and lowland dams mentioned in the text indicated. Topography derived from the Shuttle Radar Topographic Mission Digital Elevation Model, National Aeronautics and Space Administrations, USA (SRTM-DEM, NASA).

Based on their mineralogy, Gibbs [3] concluded that most sediments carried by the Amazon River are derived from the Andes (Fig 1). Current estimates suggest that 93% of all sediments in the Amazon River system are derived from this source [4]. High correlations between total suspended sediments (TSS) and particulate phosphorus and nitrogen concentrations in these rivers [5] indicate that most sediment-bound nutrients in the river system are also derived from the Andes. The damming of major tributaries draining the Andes could, therefore, reduce the supply of both sediments and nutrients to the Amazon lowlands (Fig 1).

While dam building is only beginning in the Amazon, dams have already caused significant declines in the sediment loads of other large river systems with major consequences for fluvial ecosystems and human populations downstream [6–10]. Large reductions in sediment loads following impoundment have resulted in rapid downstream channel erosion with decreases in bed elevations, changes in channel width and the loss of riparian habitats and vegetation [7, 10, 11–14]. In rivers where sediment supplies are not replenished by downstream sources, these impacts have extended to lowland floodplains and deltas where the loss of residential areas and farmlands due to increased flooding and subsidence is now a global concern [9]. Reductions in the supply of riverine nutrients to downstream floodplains, deltas and coastal areas have led to losses in soil fertility, accompanied with increased use of artificial fertilizers [8] and declines in aquatic primary production and fish yield [7, 8, 15, 16]. The changes expected below the new Andean dams will depend on the amount of sediment and nutrients retained by these impoundments and the hydrological and geomorphological dynamics of the river systems below. Downstream inputs from tributaries and channel exchange processes in the foreland and lowland regions [4, 17–20] may replenish, in part, the supply of sediments and nutrients retained by the dams before the rivers reach the lowland floodplains (Fig 1). However, if the initial decline in sediment and nutrient supplies below the dams are large and a significant part of this reduction propagates downstream, lowland environments are likely to be affected.

The seasonal flood-pulse of the Amazon River plays a fundamental role in maintaining the diversity and productivity of lowland floodplain environments [21, 22]. Inputs of river-borne nutrients during seasonal floods maintain the fertility of lowland floodplain soils [23–25] and the productivity of alluvial wetlands [26–28]. The dynamic interplay between floodplain topography and river flood cycle [29, 30] creates a complex mosaic of floodplain environments with varying flood dynamics and a diversity of aquatic flora and fauna adapted to these conditions [1, 31–36]. The production dynamics and phenology of this biota are synchronized to local inundation patterns [21, 37, 38]. Fish production and yield are linked to flood dynamics, with the highest yields occurring 1–2 years after the largest floods [40–41].

Variations in water discharge through hydroelectric turbines are normally limited by dam managers in order to stabilize power generation [11, 42]. This operational norm can have a large impact on the flood-pulse below the dam, reducing maximum stage heights and flooded areas, increasing minimum stage heights and flooded areas and altering the spatial pattern of inundation across the floodplain [11, 43, 44]. If similar changes occur below the Andean dams, they could have profound effects on the flora, fauna and human populations occupying these regions, including a reduction in floodplain fertility and fish yield, the permanent flooding and mortality of low lying vegetation and the large-scale disruption of plant phenology and growth cycles [1, 36, 45].

A different suite of impacts is expected upstream of the Andean dams, as fluvial ecosystems are transformed into reservoirs. The transformation of a flowing river and its accompanying upland basin into a large lake results in the destruction of terrestrial vegetation and major changes in the structure and functioning of the aquatic ecosystem [1, 46]. The generally well mixed riverine water column becomes thermally stratified in the reservoir. Fine sediments and sands, which were suspended in the river, decant in the reservoir, causing the siltation of benthic environments [13]. As inundated terrestrial vegetation dies and decomposes, dissolved oxygen levels fall and significant amounts of dissolved organic matter, nutrients and greenhouse gases (GHGs:CO_2_ and CH_4_) are released to the overlying water and atmosphere [47–49]. These conditions favor the methylation of dissolved inorganic mercury (MeHg), originally present in the inflowing river, and its bioaccumulation in fish and other aquatic organisms [50, 51]. GHGs and MeHg produced in reservoir bottom waters are also exported to the fluvial ecosystem below the dam, contributing to increased GHG emissions and mercury contamination in these regions [47, 48, 52, 53]. Increased nutrient concentrations from internal and external loading together with improved light penetration generally result in elevated levels of primary production and fish yield in reservoirs, which can lead to the development of important local fishing industries, especially in tropical reservoirs [54, 55]. Elevated fish production is expected in the new Andean reservoirs due to large inputs of nutrients from Andean tributaries [56]. This is likely to bring economic benefits to these regions but may also promote mercury contamination in the populations that consume these fish.

Several recent attempts have been made to evaluate the potential impacts of hydropower development on the Amazon [1, 2, 9, 36, 57, 58] and other large tropical river ecosystems [9, 57, 59]. These analyses have focused on aquatic and terrestrial biodiversity [36, 57, 59], sediment dynamics [9] or integrated multiple impacts [1, 2, 58]. They have generally been qualitative analyses, at most comparing regional distributions of dams with maps of species richness or habitats [57, 59]. The exception was a recent analysis of Labtrubesse *et al*. [58], which used spatial indexes, based on quantitative estimates of current land use cover, sediment yield, hydrological variability, geomorphological variability, and fluvial connectivity, to evaluate current and future vulnerability to hydropower development across the Amazon Basin. While this analysis attributed the highest vulnerability to basins draining the Andean headwaters, the exact nature and magnitude of these impacts were not examined. Quantitative predictions of these impacts, based on mechanistic and process-based associations with dam construction, are needed.

Here we use historical data from Amazonian rivers and reservoirs, together with mechanistic scenarios to examine the potential impacts of six planned Andean dams on Amazon fluvial ecosystems. We consider impacts both above and below the dams, including 1) the reduction in downstream sediment supply, 2) the reduction in downstream nutrient supply, 3) changes in downstream flood pulse, 4) changes in upstream and downstream fish yield, 5) reservoir siltation, 6) greenhouse gas emissions above and below the dams, and 7) mercury contamination above and below the dams. The analysis provides quantitative predictions of the magnitude and extent of these impacts along with descriptions of the methods and relationships used to generate them.

## Materials and methods

**Fig 2:**
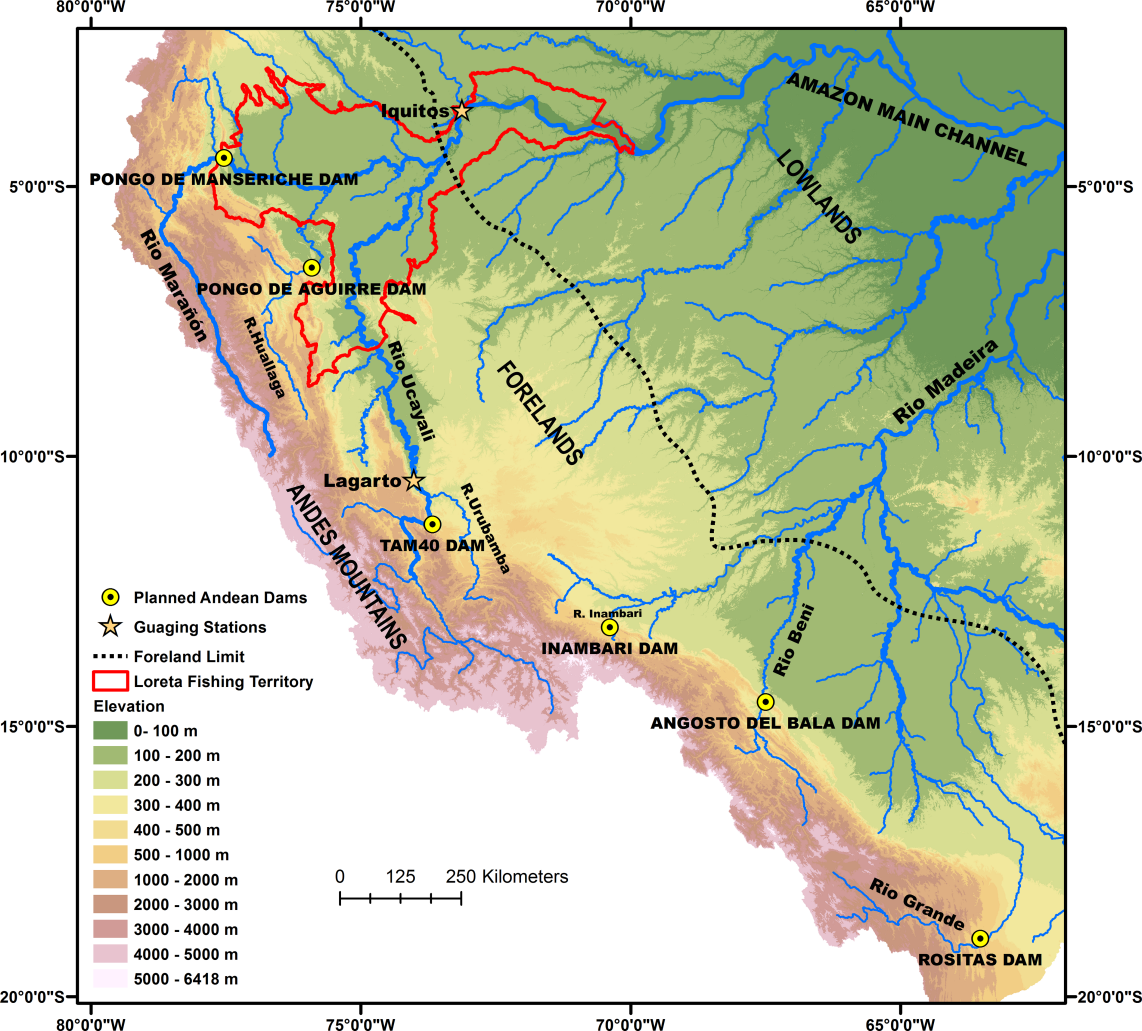
Locations of planned Andean dams and the Loreto fishing territory. Impacted tributaries, relevant gauging stations and key geomorphological features are indicated. Topography derived from NASA, SRTM-DEM [60].

This analysis considers the potential impacts of six hydroelectric dams planned for construction on major tributaries draining the Andean highlands: Pongo de Manseriche Dam on the Marañón River, Pongo de Aguirre Dam on the Huallaga River, TAM 40 Dam on the Ucayali River, Agosto del Bala Dam on the Beni River, Inambari Dam on the Inambari River and Rosita Dam on the Grande River (Fig 2). The expected characteristics of these dams and their associated storage reservoirs are indicated in Table 1.

**Table 1:** Expected characteristics of six planned Andean dams.

Dam	Country	River	MW^a^	Basin Area^b^, 10^3^ km^2^	Reservoir Area^b^, km^2^	Reservoir Volume^b^, 10^8^ m^3^	Inflow, 10^8^m^3^y^-1^
Angosto del Bala	Bolivia	Beni	1600	69.8	1,048	200	646^c^
TAM 40	Peru	Ucayali	1286	123.6	n.a.	n.a.	1,337^d^
Pongo de Aguirre	Peru	Huallaga	750	69.4	n.a.	n.a.	941^d^
Pongo de Manseriche	Peru	Marañón	4500	114.2	7204	7,420	1,582^d^
Rositas	Bolivia	Grande	400	59.2	241	125	95^c^
Inambari	Peru	Inambari	1500	18.3	319	158	487^e^

^a^ [2, supplemental data].

^b^ estimated here, using dam location, specified reservoir surface elevation and SRTM–DEM [60]; water inflow = measured discharge at dam site.

^c^ [18].

^d^ [61], inflow for TAM40 dam estimated from discharge measured at Lagartos gauging station, corrected by ratio of basin areas above these sites, assuming similar runoff.

^e^ B. Forsberg, unpublished data

n.a. = not available.

All six dams will be located in the Andes Mountains near the forelands (Fig 2). Together, their associated tributaries drain 436,000 km^2^, about 69% of the Andean highlands, defined here as all area in the western and south-western Amazon basin above 500 m elevation. All are white water rivers with elevated concentrations of nutrient-rich suspended sediments, derived from weathering and erosion in the Andean highlands [3, 5, 18, 61]. Based on observations of these and rivers of similar elevations and geomorphological contexts [62], we expect them to transport bed loads of silt, sand, gravel and cobbles. Below the dam sites, these rivers flow onto the Sub-Andean forelands, a geomorphologically dynamic region characterized by high rates of channel erosion, deposition and lateral migration (Figs 1 and 2) [17, 18, 20, 63–66]. Below this region, the tributaries flow through the Amazon lowlands, eventually reaching the Amazon delta and estuary. The lowlands are characterized by large exchanges of sediment between floodplains and river channels with large net accumulations of sediments occurring along the central floodplains and delta [19].

### Impact on the downstream sediment supply

The expected impact of the six Andean dams on downstream sediment supply was determined from the technical specifications of the dams and existing information on their tributary rivers (Table 1) [5,18, 61] The reservoirs created by these dams will have large volumes relative to their inflows which will result in long hydraulic residence times and high sediment trapping efficiencies [67]. Only four of the proposed dams had all of the information necessary to calculate sediment trapping efficiency. Sediment trapping efficiencies (T, %) for these dams were estimated using a mathematical representation of Brune’s Curve (67):

$$T=100\times {\left[0.97\right]}^{0.19^{\left(V÷I\right)}}$$ (1)

where, T = trapping efficiency, %

V = reservoir storage volume, m^3^, and
l = annual water inflow to the reservoir, m^3^

The trapping efficiencies for Angosto del Bala, Pongo de Manseriche, Rosita and Inambari dams, determined with this equation, were 93, 98, 97 and 93%, respectively. Given the proximity of these values to 100% and the errors associated with the measurements used to derive Brune’s original relationship, we assumed that all suspended sediment and bedload will be retained by these dams. This will definitely be true for the coarse suspended sediment and bedload fractions which will have the greatest impact on downstream channel geomorphology. Given the similarity of the inflows, reservoir design and geomorphological contexts of the remaining two dams, we assumed that they will retain 100% of the inflowing sediment load. The reduction in suspended sediment discharge following impoundment was thus assumed to be equivalent to the annual sediment discharges reported for these rivers near the planned dam sites [18, 61, 62] (B. Forsberg, unpublished).The reduction in bedload discharge was then added to these values, assuming that bedload represents 10% of total sediment discharge before impoundment. This average bedload contribution was obtained from Guyot [62] who reviewed existing estimates of bedload transport in the Andean region. Sediment discharge for the TAM 40 site was estimated from the value measured at Lagartos [61], corrected by the ratio of basin areas above these sites, assuming similar sediment yields per unit area.

The expected decline in Andean sediment yield due to the dams was estimated from the percent of the Andean highlands drained by the six dam basins, assuming that sediment yield per unit area (tons km^-2^ y^-1^) from this region was relatively constant. The total area of Andean highlands with elevation >500 m and the total area of these highlands drained by the six dam basins were calculated from the SRTM-DEM [60]. The proportion of the Andean highlands drained by the dams and the percent decline in Andean sediment yield were estimated from the ratio of these values. The expected decline in sediment supply for the entire Amazon due to the dams was estimated from the % reduction in Andean yield, assuming the Andes accounts for 93% of the basin-wide yield [4].

### Impact on the downstream nutrient supply

We used nutrient and sediment concentrations for the Amazon River and its main lowland tributaries, determined by the CAMREX Project [5] (http://dx.doi.org/10.3334/ORNLDAAC/904), together with the sediment reductions determined above, to estimate the decline in nutrient supplies expected below the six Andean dams following impoundment. The sampling strategy of CAMREX was to obtain flow-weighted samples of water and sediments for chemical analysis at 18 fluvial sites along a 2000 km reach of the lowland Amazon mainstem, including major tributaries, during 13 cruises between 1982 and 1991 at different stages of the hydrographic cycle. A single positive linear regression passing through the origin was found to describe the relationship between particulate phosphorus (PP) and total suspended sediment (TSS) concentrations for all rivers sampled [5] (S1 Fig). The average P content of sediments, derived from the slope of this relationship, 0.08% (w/w), was assumed to apply to suspended sediments at the dam sites. A negative non- linear relationship was found between percent particulate nitrogen (%PN) and TSS concentrations in Amazon rivers that approached an asymptotic value of 0.097% (w/w) at TSS levels above 400 mg L^-1^ (S2 Fig). Since the average TSS levels at the proposed Andean dam sites, estimated by dividing annual sediment load by annual water discharge were all above 400 mg L^-1^, the nitrogen content in their sediments was assumed to be 0.097%. The concentrations of total dissolved nitrogen (TDN, mg m^-3^) and total dissolved phosphorus (TDN, mg m^-3^) did not vary systematically with TSS or any other parameter measured. McClain *et al*. [68] also found the levels of dissolved nitrogen and phosphorus in Andean head water tributaries to be similar to those in Amazon lowland rivers. The average concentrations of TDN and TDP determined by CAMREX, 325 and 39 mg m^-3^, respectively, were therefore used to represent the dissolved nutrient concentrations in Andean tributaries near the dam sites.

PP and PN fluxes below the six dam sites before impoundment were estimated by multiplying the %P and %N contents of sediments from CAMREX by the annual discharge of suspended sediments measured at the dam sites [18, 61, 62] (B. Forsberg, unpublished). PP and PN fluxes below the dams after impoundment were considered to be zero, assuming that all particulates were retained by the dam [67]. Bedload fluxes were not included in this calculation. The downstream fluxes of dissolved nutrients both before and after impoundment were estimated by multiplying the average TDN and TDP concentrations from CAMREX by annual water discharges measured at the dam sites (Table 1), assuming that dissolved nutrient fluxes would not change following impoundment. The expected reductions in TN and TP were calculated from the difference between the sum of the particulate and dissolved fluxes before and after impoundment, and, due to the assumptions used, reflect only changes in the particulate fluxes.

The reduction in TP and TN yields from the Andes Region due to the dams was estimated using the mean composition of the nutrient loads determined above, assuming that particulate nutrients will decline in the same proportion as sediments. The reduction in total nutrient supplies to the entire Amazon was estimated by dividing the sum of the nutrients retained by the six dams by the total nutrient yield for the Amazon Basin, including both particulate and dissolved components. Particulate nutrient yields for the Amazon were estimated from the sum of particulate nutrients trapped by the 6 dams divided by the product of the proportion of Andean sediments trapped by the dams and the proportion of Andean sediments in the Amazonian yield (approximately 0.93 [4]). Total dissolved nutrient yields for the Amazon were estimated from the product of the average discharge of the Amazon River at its mouth (205,000 m^3^ s^-1^ [69]) and the average concentration of total dissolved nutrients determined by the CAMREX Project for Amazon tributaries (TDN = 325 mg m^-3^; TDP = 39 mg m^-3^).

The current supply of PP to the central floodplain and delta plain regions was estimated by multiplying the combined annual accumulation of sediment in these regions [19] by the average phosphorus content of Amazon River sediments (0.08%) [5]. The input of PN was estimated in a similar way using the average N content (by weight) of river sediments along the central Amazon floodplain determined by the CAMREX project (0.11%, B. Forsberg, unpublished data). The reduction in these supplies following dam construction was presumed equivalent to the basin-wide drop expected for sediments.

### Impact on the flood pulse

**Fig 3:**
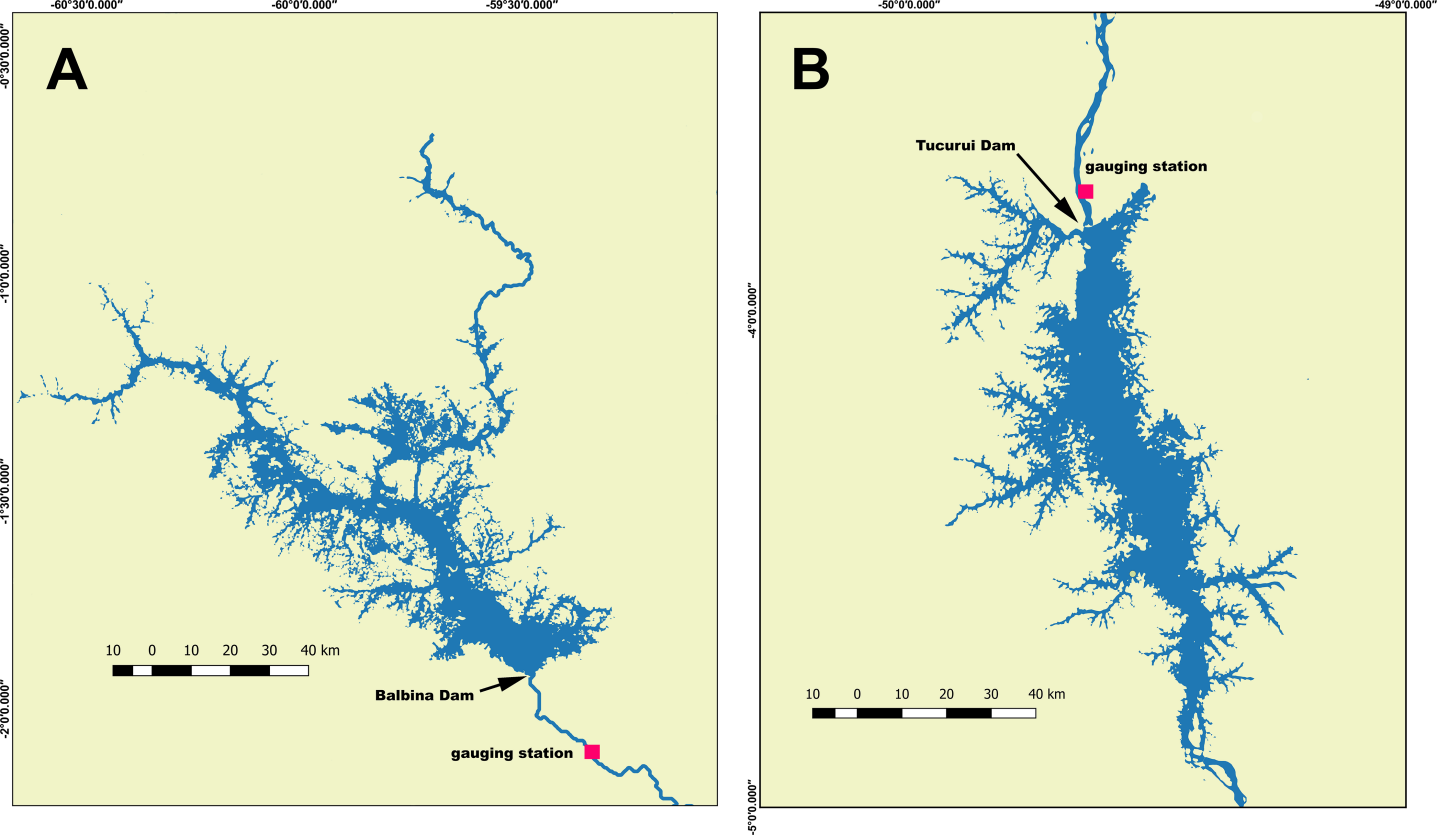
**Locations of A) Balbina Dam on the Uatumã River and B) Tucurui Dam on the Tocantins River, showing reservoirs and locations of downstream gauging stations.** Cachoeira Morena and Tucurui gauging stations are indicated below the dams. Station locations from the Brazilian National Water Agency, ANA [70]. Reservoir area derived from NASA, SRTM-DEM and LANDSAT imagery [60].

The impact of impoundment on the flood pulses downstream from the Andean dams will depend on the specifications of each impoundment, the hydrological and geomorphological characteristics of the associated rivers and the discharge management regime (DMR) adopted at each dam. DMRs were not specified for any of these planned dams. Historical stage height and discharge records, necessary to characterize hydrological variability, were also unavailable for the rivers at the dam sites. Insight into the nature of these impacts was therefore obtained by examining historical changes in stage height below the two largest existing hydroelectric storage dams in the Amazon, Balbina and Tucurui (Figs 1 and 3). Balbina Dam was built on the Uatumã River in 1987, creating a storage reservoir with an average surface area of 1770 km^2^, an average depth of 10 m and an installed power generating capacity of 250 MW [48]. The volume of the reservoir is 177 x 10^8^ m^3^, the annual discharge is 180 x 10^8^ m^3^ and the trapping efficiency of the dam, estimated with Eq 1, is 99% [48]. Tucurui Dam was built on the Tocantins River in 1985, creating a storage reservoir with a surface area of 2,875 km^2^, an average depth of 19 m and a current installed generating capacity of 8370 MW. The reservoir has a volume of 455 x 10^8^ m^3^, an annual discharge of 3470 x 10^8^ m^3^ and a sediment trapping efficiency of 98%. The storage volume, annual inflow (estimated from discharge) trapping efficiency and ratio of volume to inflow of both of these reservoirs are all within the range of values expected for the Andean dams, with the exception of the Manseriche which will have an exceptionally high volume: inflow ratio (Table 1). Thus, if similar flow management schemes are employed, changes in stage and discharge variation below the Andean dams are likely to be similar. Changes in the characteristics of the flood pulse below Balbina Dam were evaluated by comparing average monthly stage heights and annual flood periods at specific stage heights, measured at the Cachoeira Morena gauging station, 30 km downstream from the dam [70] (Fig 3), before construction began (1973–1982) and after the dam was inaugurated (1991–2011). Changes in flood pulse below Tucurui Dam were evaluated by comparing similar stage variations at the Tucurui gauging station, 10 km downstream from the dam [70] (Fig 3), before construction began (1969–1975) and after the dam was inaugurated (1991–2011). Changes in mean monthly stage height were used to identify elevations on the associated river floodplain that became permanently flooded or permanently dry following impoundment, while changes in flood period at all stage heights were used to evaluate inundation changes at all elevations on the floodplain.

### Impact on downstream fish yield

The impact of changes in peak maximum and minimum flooded area on fish yield downstream from the dams planned for the Ucayali, Marañón and Huallaga rivers was investigated for the territory exploited by commercial and riparian community fishermen in the Loreto region of Peru (Fig 2). This area is composed of a complex matrix of river channels, and seasonally inundated alluvial floodplains. Fish production in the region is sustained by high levels of primary production on floodplains supported by river-borne nutrients. Data for annual fish yield were obtained from DIREPRO (Dirección Regional de Producción, Peru) for the main cities along the Amazon, Ucayali and Marañón floodplains between 2004 and 2011 and was organized by fishing zone, fishing fleet and species (S1 Table). Total annual yield included all fish caught in the fishing zones located in the fishing territory delimited in Fig 2.

Daily flooded areas for the Loreto fishing territory were estimated for a 12 year period (1998–2009), using the MGB-IPH large-scale numerical hydrological model, that was developed and validated specifically for the Amazon Basin [71, 72]. Maximum annual flooded areas (MxFA) and minimum annual flooded areas (MiFA) for the period 1999–2011, that included 5 years before the beginning of the fish yield record and 2 years after the simulated data set, were estimated from a linear regression between simulated daily flooding in the Loreto fishing territory and daily stage height measured at Iquitos, (SENAMI, Peru) (S1 Table).

Inter-annual variation in fishing effort has been shown to be an important predictor of fish yield in the Amazon [73]. While precise information on fishing effort was unavailable for the major fishing zones [74, 75], the total number of fishing zones (FZ) varied between years and was used as a proxy for fishing effort in the analysis. Historic variations in fish yield, fishing zones, minimum flooded area and peak flooded area for the Loreto Region are provided in S1 Table. The influence of MxFA, MiFA and FZ on annual fish yield was investigated with simple and multiple linear regression analyses, using time lags of 0–5 years to evaluate the delayed effects of flooding, as suggested by Welcomme [39]. The 10 year average maximum inundated area was then used together with the best regression model to predict the effect of proportional reductions in mean maximum flood extent, due to impoundment, on fish yield.

### Impact on upstream fish yield

Inputs of nutrients from inflowing Andean tributaries are expected to dominate nutrient loading to the six planned reservoirs. The levels of total nutrients and aquatic production in these systems are therefore expected to be similar to those encountered in Amazon floodplain lakes associated with Andean tributaries. The dominant primary producer in these deep, steep-sided reservoirs is expected to be phytoplankton. Forsberg *et al*. 2017 [76] estimated average daily integral gross production for phytoplankton in Amazon floodplain lakes associated with Andean tributaries to be 3 g O_2_ m^-2^ d^-1^.This value was used, together with the empirical relationship developed by Melack [77] for tropical lakes, to estimate the fish yield per unit of area in the Andean reservoirs:

$$Log({TY}_{a})=0.122PG+0.95$$ (2)

where, TY_a_ = annual areal fish yield, kg ha^-1^ y^-1^, and

GP = gross integral daily phytoplankton production, gO_2_ m^-2^ d^-1^

Total annual fish yield was estimated by multiplying the areal fish yield, determined with Eq 2, by the predicted reservoir surface area, available for the Angosta del Bala, Pongo de Manseriche, Rositas and Inambari reservoirs (Table 1).

### Reservoir sedimentation

The information necessary to estimate reservoir sedimentation rates was available for the Angosta del Bala, Pongo de Manseriche, Rositas and Inambari reservoirs. Storage volumes were estimated from specified dam locations and reservoir surface heights [78], using the SRTM—DEM [60] (Table 1). All reservoirs will be a deep and steep sided, with average depths ranging from 19–103 m. The number of years required to completely fill each reservoir was estimated by dividing the storage volume by the volume of sediments delivered to the system annually. The volume of suspended sediments reaching the reservoir was estimated by dividing the annual sediment discharge (by weight) near the proposed dam site [18, 61] (B. Forsberg, unpublished) by the bulk density of recently deposited Amazon sediments, estimated by Devol *et al*.[79] to be 1,396 kg m^-3^. The additional contribution of bedload to sediment volume was estimated assuming that bedload transport was 10% of total sediment discharge [62] and that the bulk density of bed material was similar to that of cobblestones, estimated by Carling and Reader [80] to be 1890 kg m^-3^.

### Greenhouse gas emissions

Information necessary to model greenhouse gas emission was available for the Angosta del Bala, Pongo de Manseriche, Rositas and Inambari reservoirs. These reservoirs are expected to receive high concentrations of nutrients from their associated Andean tributaries and inundate extensive areas of terrestrial vegetation (Table 1). As in other nutrient-rich tropical reservoirs [48, 81, 82], they are expected to be productive and generate significant quantities of greenhouse gases both above and below their respective dams. Potential emissions of CO_2_ and CH_4_ (CO_2_ flux, CH_4_ flux, mgC m^-2^ d^-1^) from reservoir surfaces, by both diffusion and bubbles, were estimated using the following empirical relationships, derived from data for 85 globally distributed reservoirs [82]:

$$Log({CO}_{2}flux+400)=3.06-0.16Log(age)-0.01Lat+0.41Log(DOC)$$ (3)

$$Log({CH}_{4}flux)=1.33-0.36Log(age)-0.32Log(mean\;depth)+0.39Log({DOC}_{Load})-0.01Lat$$ (4)

where,

DOC = dissolved organic carbon concentration in the reservoir, mgC L^-1^,
DOC_Load_ = DOC loading to the reservoir, mgC m^-2^ d^-1^,
age = years since impoundment,
mean depth = the mean depth of the reservoir in meters, and
Lat = mean latitude of the reservoir

External DOC loading was estimated from the product of tributary inflow and DOC concentration divided by reservoir area. DOC concentrations were obtained from the ORE-HYBAM project (www.ore-hybam.org) or from an empirical relationship between DOC and elevation developed for Andean tributaries [83]. External DOC loading and background DOC concentrations were assumed to be constant over time. Additional internal DOC loading, derived from the decay of inundated terrestrial vegetation, was assumed to decline gradually following impoundment as the terrestrial carbon stock diminishes. The initial areal carbon stock of terrestrial vegetation in all reservoir areas was assumed equivalent to that estimated by Vega *et al*. [84] for the Inambari Reservoir, 30,300 tons C km^-2^. Total terrestrial carbon stock was estimated by multiplying this value by the projected surface area of each reservoir. This carbon stock and internal DOC loading was assumed to decline over time at an exponential decay rate of 0.23 y^-1^, similar to the rate of decline in emissions observed at Petit Saut Reservoir, French Guyana, after impoundment [47]. This variable loading rate was used, together with the estimates of upstream loading and background DOC, to calculate the change in DOC, DOC_Load_ and greenhouse gas emissions over time. Methane emissions were converted to CO_2_-equivalents (CO_2e_) assuming a 100 year global warming potential (GWP) for methane of 34 [85].

Significant quantities of greenhouse gases are also released downstream from dams [47, 48, 86, 87]. These fluxes include both degassing at the turbine outflow and diffusive emissions from the downstream river. The total downstream flux has been shown to be proportional to the power generating capacity of a dam [87] and to its turbine discharge [88]. The downstream emissions of CO_2_-equivalent carbon for Balbina and Petit Saut dams, normalized to turbine discharge, were 22.6 and 26.9 gC-CO_2e_ m^-3^, respectively, with an average value of 24.8 gC-CO_2e_ m^-3^ [88]. This average value was multiplied by the annual discharge of each reservoir to estimate the initial CO_2_ equivalent carbon emission expected downstream of the dams. Since downstream emissions are fueled, in large part, by internal carbon loading to a reservoir [47], an exponential decay rate of 0.23 y^-1^ was also applied to these initial values to simulate the expected decline over time.

In order to compare emission characteristics between dams and between these planned hydroelectric facilities and alternative energy sources, we estimated Carbon Emission Factors (CEFs) for each dam. These factors were estimated for each dam by dividing the total annual CO_2_ equivalent carbon emission in tons C-CO_2_e, including both reservoir and downstream fluxes, averaged over the first 30 years of dam operation, by the total annual power generation of the dam in MWhrs.

### Mercury dynamics

Empirical models have been developed for tributaries of the Amazon relating mercury levels in fish and human hair to trophic level, pH, DOC and wetland densities [89–91]. However, these relationships vary geographically [92], and there are insufficient data for Andean tributaries to develop specific relationships for this region. The dynamics of mercury in reservoirs is also different than that found in free flowing river systems, due to the anoxic conditions, expected following impoundment, that promote high rates of mercury methylation and subsequent biomagnification. Relationships between the levels of MeHg in water or fish and terrestrial carbon stocks or percent flooding relative to volume and have been developed for some north temperate reservoirs [93, 94], but similar relationships are unavailable for tropical systems. Due to the lack of mechanistic models for predicting Hg dynamics in the Andean dams, we were limited to examining the temporal variation of Hg contamination in Balbina (Fig 3), the only Amazonian reservoir with a consistent historical time series of mercury measurements. There was no gold mining or other anthropogenic sources of mercury, besides regional atmospheric inputs, in the drainage basin upstream of Balbina Dam, during the period considered. Mercury present in the system is presumed to be predominantly of natural origin. Historical data for Balbina Reservoir included measurements of total mercury in *Cichla* spp., the main fish predator in the system (B. Forsberg, unpublished data), and total mercury in the hair of fish-eating human populations that historically exploited this resource [95]. Fish of varying sizes were collected between 1992 and 2003, while Hg levels in hair were obtained for the period of 1995–2000 [95]. For the unpublished fish data, boneless and skinless samples of dorsal muscle were collected and stored frozen until analysis. Following digestion these samples were analyzed for total mercury by cold vapor atomic absorption spectrophotometry [96, 97]. Mean Hg concentrations in fish were normalized to a standard size.

## Results

### Impact on the downstream sediment supply

The combined reduction in sediment discharge expected for all six rivers was estimated at 894 x 10^6^ tons y^1^ (Table 2). The estimated area of all Andean highlands with elevation >500 m and the total area of these highlands drained by the six dam basins were 628,000 km^2^ and 436,000 km^2^, respectively. The percent of the Andean highlands drained by the six dam basins and the expected decline in Andean sediment yield, represented by the above value, is therefore 69%. Since export from the Andes accounts for approximately 93% of all sediment yield in the Amazon basin [4], the construction of these six dams is expected to result in a 64% reduction of in the basin-wide sediment supply.

**Table 2:** Predicted reduction in sediment discharge below six planned Andean dams following impoundment.

Dam	Predicted reduction in sediment discharge,10^6^ t y^-1^
Angosto del Bala	243^a^
TAM 40	272^b^
Pongo de Aguirre	57^b^
Pongo de Manseriche	166^b^
Rosita	138^a^
Inambari	18^c^
TOTAL	894

Estimates assume that 100% of suspended and bedload sediments are retained by all dams.

^a^ [18].

^b^ [61].

^c^ B. Forsberg, unpublished data.

### Impact on the downstream nutrient supply

The combined reduction in the discharge of TP and TN for all six dams was approximately 0.6 and 0.8 x 10^6^ tons y^-1^, corresponding to a decline of 97% and 83%, respectively (Table 3). The larger proportional drop in TP reflected a higher percentage of particulates and resulted in an increase in the average ratio of TN:TP in the nutrient load from 1.4:1 to 8.6:1.

**Table 3:** Predicted reduction in nutrient fluxes below six Andean dams after impoundment.

Dam	Current fluxes 10^6^ tons y^-1^		With dam 10^6^ tons y^-1^		Reduction 10^6^ tons y^-1^		% change	
	TP	TN	TP	TN	TP	TN	TP	TN
Angosto del Bala	0.177	0.233	0.002	0.021	0.175	0.212	-99	-91
TAM 40	0.201	0.280	0.005	0.043	0.196	0.237	-98	-85
Pongo de Aguirre	0.044	0.080	0.003	0.030	0.041	0.049	-92	-62
Pongo de Manseriche	0.125	0.195	0.006	0.051	0.119	0.144	-95	-74
Rosita	0.100	0.124	0.000	0.003	0.100	0.121	-100	-98
Inambari	0.015	0.031	0.002	0.016	0.013	0.016	-88	-50
TOTAL	0.662	0.944	0.019	0.164	0.643	0.780	-97	-83

Estimates assume 100% retention of particulate fractions and no retention of dissolved fractions. Sources for data used in calculations: [18, 61, 5], B. Forsberg, unpublished data, CAMREX PROJECT (http://dx.doi.org/10.3334/ORNLDAAC/904).

Nutrient retention by these six Andean dams is expected to reduce the total supply of TP and TN from the Andean region by 67 and 57%, respectively, and reduce the supply to the entire Amazon basin by 51 and 23%. The lower impact for nitrogen reflects the greater proportion of dissolved inorganic and organic components in TN than in TP. In the basin-wide analysis, dissolved components accounted for 20% and 63% of the supplies of TP and TN, respectively. The current supplies of PP and PN to the central floodplain and delta plain were estimated as 4–5 x10^5^ and 6–7 X 10^5^ tons y^-1^, respectively. These fluxes are expected to decline by 64%, about 3 x 10^5^ and 4 x 10^5^ tons y^-1^, respectively, if the dams are built.

### Impact on the river flood pulse

**Fig 4:**
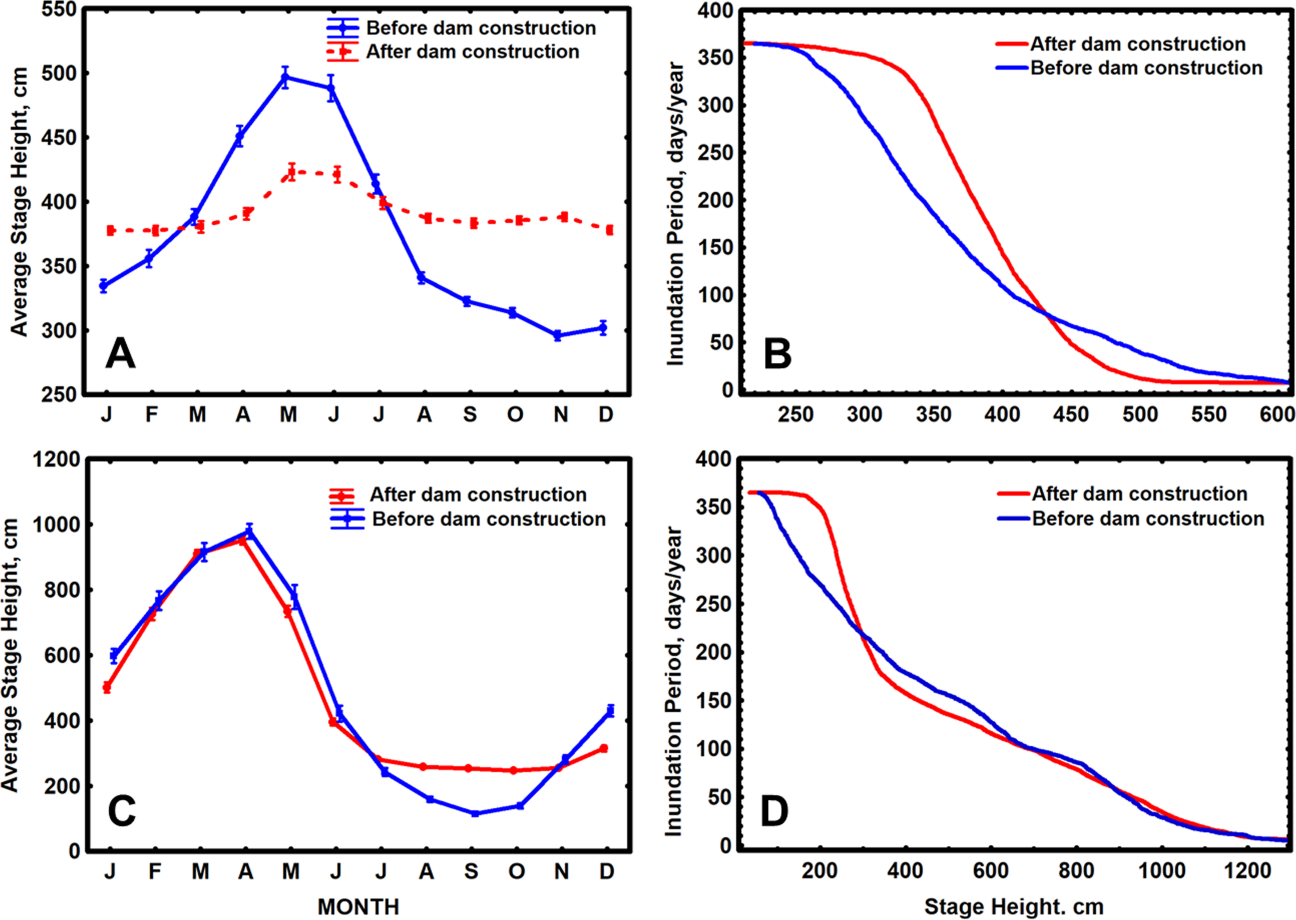
**Changes in average monthly stage heights and inundation periods for stage heights of the Uatumã River below Balbina dam (A and B, respectively) and of the Tocantins River below Tucurui dam (C and D, respectively), following impoundment**. Pre-construction periods (blue line) = 1973–1982 for Balbina and 1969–1975 for Tucurui; post impoundment periods (red line) = 1991–2011 for Balbina and 1985–2014 for Tucurui. Stage data obtained from Brazilian National Water Agency, ANA [70]. Standard error bars indicated.

Both the Uatumã River below Balbina Dam and the Tocantins River below Tucurui Dam had a similar reduction in the range of mean monthly stage heights below the dams (Fig 4A and 4C), with reductions of 154 cm and 146 cm encountered for the Uatumã and Tocantins, respectively. However the magnitude of these changes relative to the Pre-impoundment Stage Range (PSR = 200 cm for the Uatumã and 869 cm for the Tocantins) and the associated seasonal patterns differed. The percent change in mean stage height relative to PSR was higher for the Uatumã (77%) than for the Tocantins (17%). An increase in average stage height was observed below both dams at low water, with maximum differences of 92 (46% PSR) and 132 cm (15% PSR) encountered for the Uatumã and Tocantins, respectively. A decrease in mean monthly stage height was observed for the Uatumã River at high water, with a maximum difference of 74 cm (37% PSR) but not for the Tocantins, where mean stage heights were similar to pre-impoundment levels. Since stage variations reflected inundation heights on the floodplains of these rivers (see discussion), these changes had important consequences for the flooding patterns in these areas. Low lying areas, below a stage height of 325 cm on the Uatumã floodplain and below 200 cm on the Tocantins floodplain, that were seasonally dry before impoundment are now almost permanently flooded, while upland portions of the Uatumã floodplain, above a stage height of 475 cm, that were seasonally flooded at high water are now almost permanently dry. If we assume a linear relationship between river stage height and inundated area for the Uatumã floodplain, the observed decline in mean stage height relative to PSR at peak high water, would have represented a 37% reduction in peak flooded area.

Changes in the general pattern of inundation on the Uatumã (Fig 4B) and Tocantins floodplains (Fig 4D) also differed. Areas above a stage height of ~430 cm on the Uatumã floodplain all had shorter flood periods while areas below this level had longer flood periods. Flood periods at all elevations except 430 cm changed significantly. Flood periods also changed at stages below ~670 cm on the Tocantins floodplain, with shorter flood periods occurring above ~280 cm and longer periods below this elevation. No significant changes in flood period were encountered at stage heights above 670 cm.

### Impact on downstream fish yield

**Fig 5:**
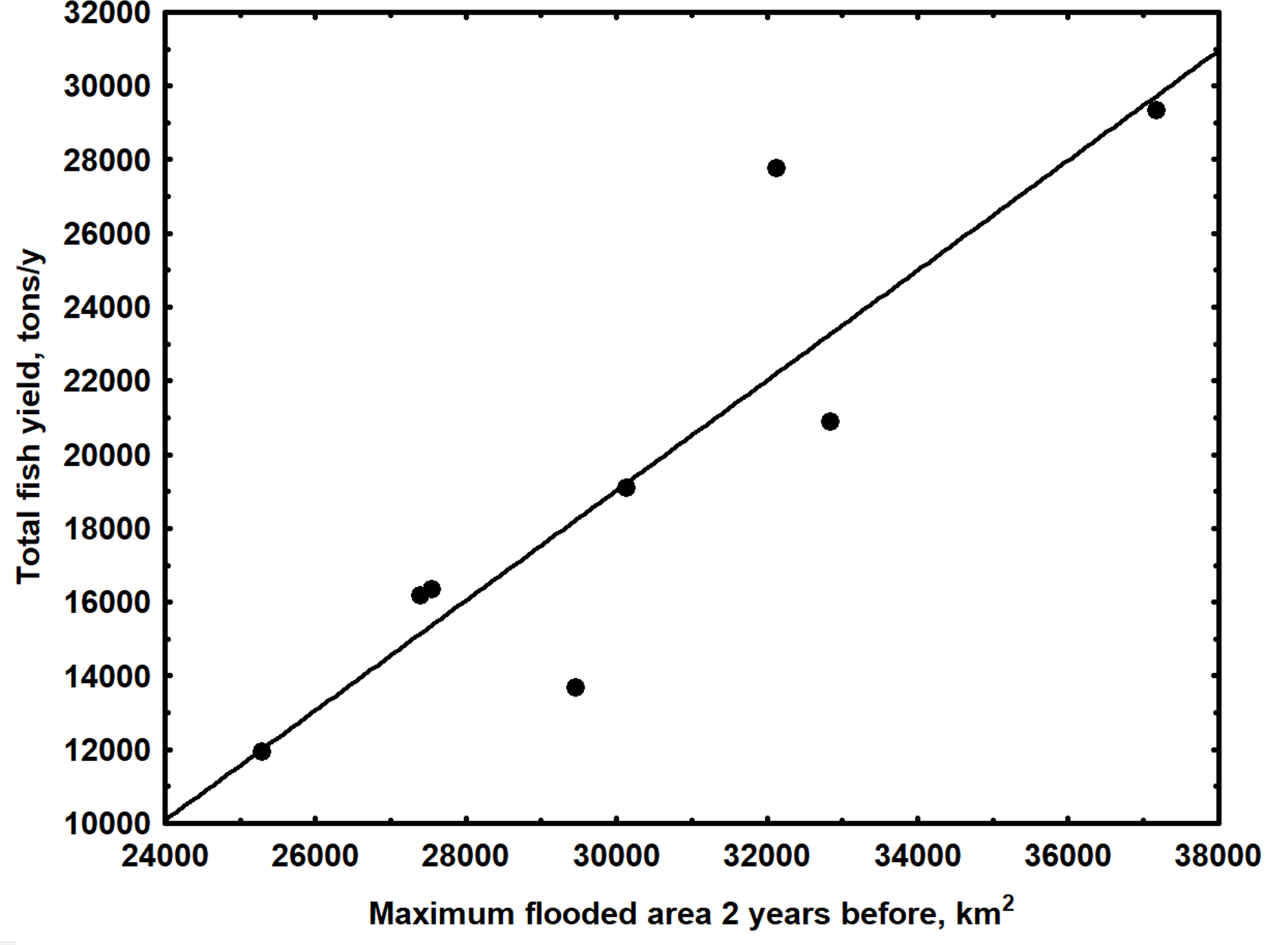
Relationship between total annual fish yield and the maximum flooded area two years earlier for the Loreto Region of Peru. Flooded areas simulated using the MGB-IPH large scale numerical hydrological model, developed specifically for the Amazon Basin [71, 72]. Annual fish yields obtained from DIREPRO (Dirección Regional de Producción, Peru).

**Fig 6:**
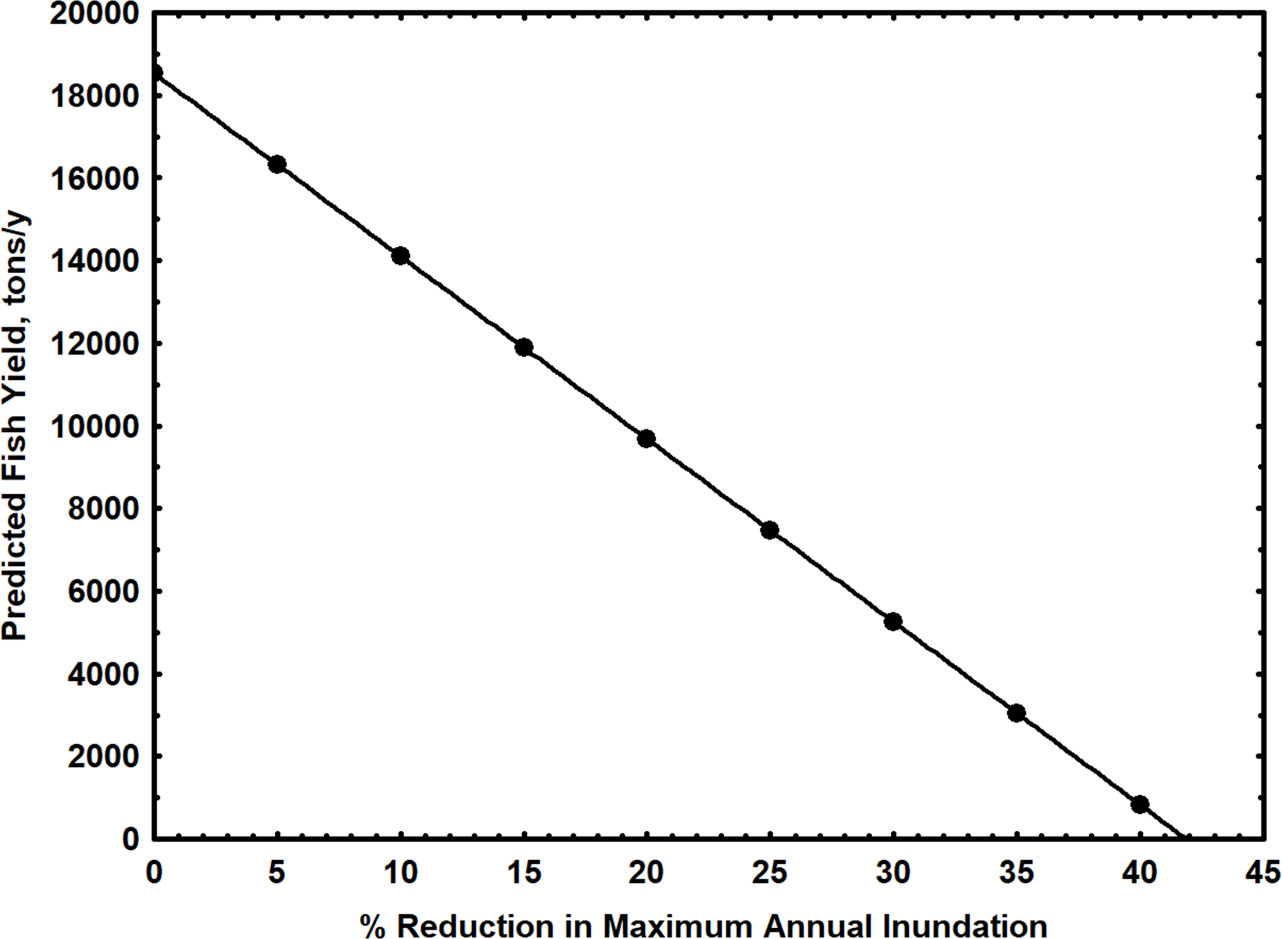
Predicted decline in annual fish yield for the Loreto Region due to a reduction in maximum annual flooded area following impoundment. Relation simulated using percentage changes in average maximum flooded area (MxFA) and regression model: *Y* = 1.5(*MxFA*) − 25,723, with a 2 year lag.

The commercial fishing fleet accounted for only 9% of the total fish yield in the Loreto region during the study period. Dispersed riparian fishing communities accounted for the remaining 91%. *Prochilodus nigricans*, *Potamorhina altamazonica* and *Psectrogaster amazonica*, small detritivore species of the Prochilodontidae and Curimatidae families, represented 55% of the total fish yield. 165 fishing zones were exploited regularly during all years, and these fishing zones accounted for 76% of the total fish yield. FZ did not contribute significantly to fish yield in a multiple linear regression analyses due to the proportionally high contribution of only a few regularly exploited fishing zones to yield (Table 4). The only significant regressions were found for both TY and YFZ against MxFA with a time of lag of 2 years (Table 4) (Fig 5). These simple linear regression models explained 75% and 83% of the observed variance in TY and YTZ for the Loreto Region, respectively. The decline in total annual fish yield predicted from the first model (Fig 5), due to a reduction in maximum inundated area following impoundment, is indicated in Fig 6.

**Table 4:** Regression models: Influence of inundation and fishing effort on annual fish yield in Loreto, considering the effect of different time lags after inundation.

Regression model	Time lag (years after inund.)/r^2^						Equation parameters, 2 year lag					
	0	1	2	3	4	5	a	s_a_	b	s_b_	c	s_c_
TY = a+b*MxFA	-	0.07	0.75*	0.26	0.07	-	-25,723	9,655	1.50	0.32		
TY = a+b*MiFA	0.15	-	-	0.03	-	-						
TY = a+b*MxFA+c*FZ	-	-	0.70*	0.14	-	-	ns	ns	1.50	0.35	ns	ns
TY = a+b*MiFA+c*FZ	0.01	-	-	-	-	-						
YFFZ = a+b*MxFA	-	0.06	0.83*	0.28	0.14	-	-22,755	6,288	1.20	0.21		
YFFZ = a+b*MiFA	0.19	-	-	0.02	-	-						

TY = total yield, tons y^-1^; YFZ = yield for regularly exploited fishing zones, tons y^-1^; FZ (fishing effort) = number of fishing zones; MxFA = maximum flooding area, km^2^ and MiFA = minimum flooding area, km^2^; Regression parameters (a, b and c) only shown for significant regressions with 2 year lag; r^2^ = % variance in yield explained by regression; s = standard error of parameter; ns = not significant.

* indicates significance at p< 0.05 level.

### Impact on upstream fish yield

The areal fish yield estimated with Eq 2 for the Andean reservoirs was 2.1 tons km^-2^ y^-1^. The total annual fish yields predicted for the 4 Andean reservoirs, multiplying this value by the expected reservoir surface areas, ranged from 509 tons y^-1^ for the Rositas Reservoir to 15,200 tons y^-1^for the Manseriche Reservoir (Table 5).

**Table 5:** Predicted fish for 4 planned Andean reservoirs.

Reservoir	Reservoir Area, km^2^	Fish yield, tons y^-1^^a^
Angosta del Bala	1,048	2,211
Pongo de Manseriche	7,204	15,200
Rositas	241	509
Inambari	319	673

^a^ Determined from estimates of phytoplankton production (FP) [76] and published relationship between FP and fish yield for tropical lakes [77].

### Reservoir sedimentation

Predicted sediment filling times for the four Andean dams ranged from 106 years for Angosta del Bala to 6240 years for Manseriche Reservoir (Table 6). The implications of these results for the reservoir environment are considered below.

**Table 6:** Predicted sediment filling times for 4 planned Andean reservoirs.

Reservoir	Fill time, years^a^
Angosta del Bala	106
Pongo de Manseriche	6240
Rositas	126
Inambari	1225

^a^ Calculated from expected reservoir volume and estimates of sediment inflow [18,61] and density [79, 80].

### Greenhouse gas emissions

Average annual CO_2_-equivalent carbon emissions, including both CO_2_ and CO_2_ equivalent CH_4_ fluxes, for reservoirs, downstream reaches and total hydroelectric systems, together with Carbon Emission Factors for the 4 planned Andean dams, were estimated for the first 30 years of operation(Table 7). Mean total emissions were lowest for Inambari Dam and highest for Manseriche Dam. Average emissions from the reservoirs were lowest for Rositas and highest for Manseriche Reservoir. Mean emissions downstream of the reservoirs were also lowest for Rositas and highest for Manseriche, and averaged 17% of mean total emissions for all dams. Carbon Emission Factors based on mean total emissions were lowest for Inambari and highest for Manseriche Dam with an arithmetic average of 0.091 tons C-CO_2_e MWhr^-1^ and a MW weighted average of 0.139 tons C-CO_2_e MWhr^-1^ for all dams.

**Table 7:** Estimated average CO_2_-equivalent carbon emissions for the reservoirs and downstream reaches of 4 planned Andean dams during the first 30 years of operation.

Dam	MW	30 year mean emission, 10^5^ tons C-CO_2_e y^-1^			Emission Factor tons C-CO_2_e MWhr^-1^^c^
		Reservoir^a^	Downstream^b^	Total	
Angosta del Bala	1600	6.4	3.8	10.2	0.072
Pongo de Manseriche	4500	71.8	9.3	81.1	0.206
Rositas	400	1.2	0.6	1.8	0.051
Inambari	1500	1.5	2.9	4.4	0.034
Total	8,000	80.9	16.6	97.5	mean 0.091

^a^ Reservoir emissions estimated following [82].

^b^ Downstream emissions estimated following [88].

^c^ Carbon Emission Factors estimated from mean total CO_2_ equivalent emission and specified power generation.

**Fig 7:**
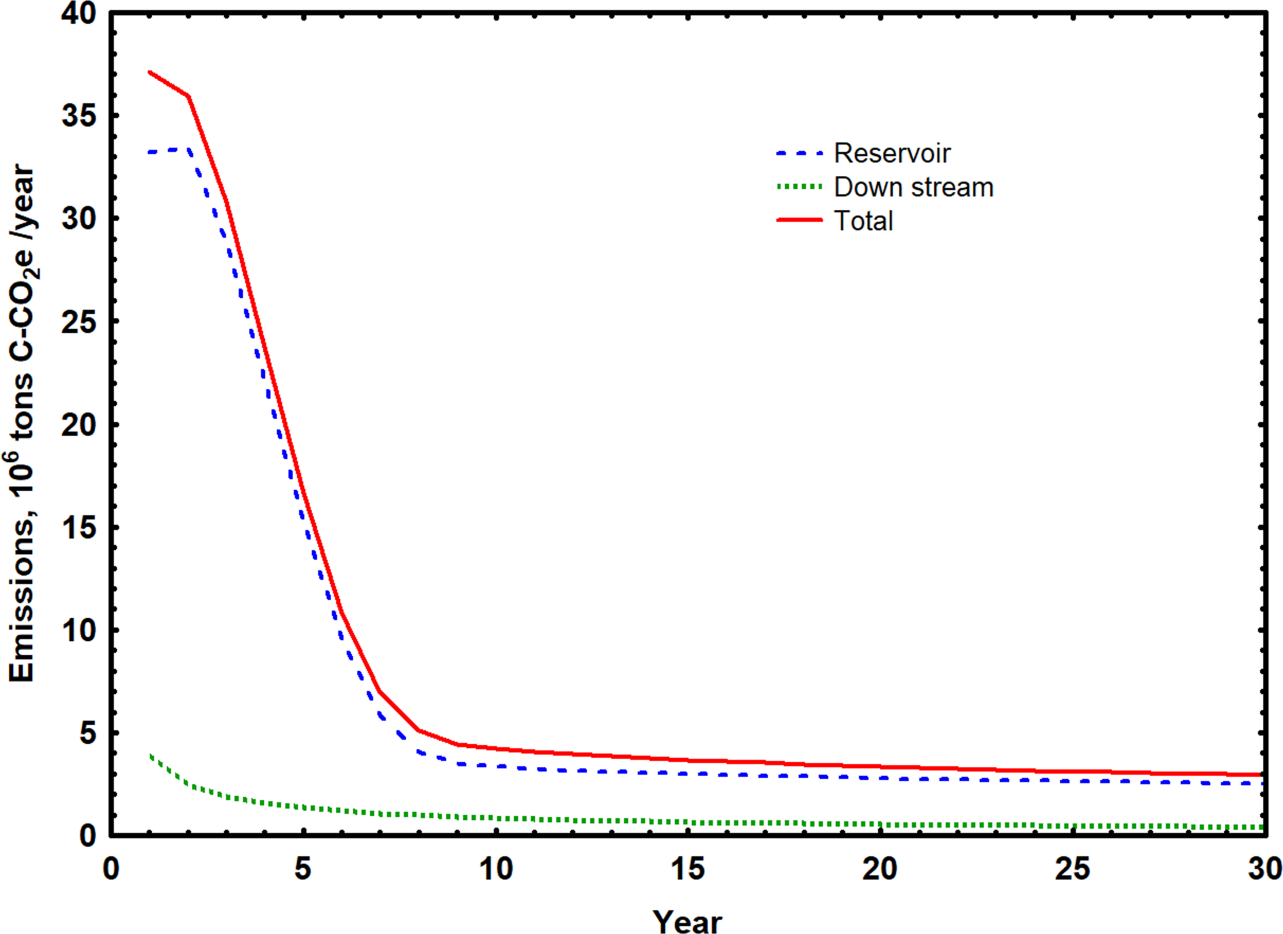
Predicted emissions of CO2-equivalent carbon upstream and downstream of Manseriche Dam during the first 30 years of operation. Reservoir emissions estimated following [82]. Downstream emissions estimated following [88].

Predicted annual emissions above and below all dams declined during the first 10 years of operation and then remained relatively stable, as illustrated in results from Manseriche Dam (Fig 7). Emissions from Manseriche Reservoir were predicted to decline from an initial high of 33.2 X 10^6^ to 2.5 X 10^6^ tons C-CO_2_e y^-1^ 30 years after impoundment. Downstream emissions were significantly lower, declining from an initial high of 3.9 X10^6^ to 0.4 X10^6^ tons C-CO_2_e y^-1^over the next 30 years. Total emissions declined from an initial high of 37.1 X 10^6^ tons C-CO_2_e y^-1^ to a rate of 2.8 X 10^6^ tons C-CO_2_e y^-1^ after 30 years. The total cumulative CO2-equivalent carbon emission for Manseriche Dam estimated for the first 30 years after impoundment was 237 X 10^6^ tons C-CO2e, with 88% released from the reservoir surface and 12% released downstream of the dam. The mean daily areal CO_2_-equivalent carbon emission rates estimated for Angosta del Bala, Manseriche, Rositas and Inambari reservoirs for the 30 period were 2,660, 3,090, 2,020 and 3,790 mg C-CO2e m ^-2^ d ^-1^, respectively, with an average value for all reservoirs of 2,890 mg C-CO2e m ^-2^ d ^-1^.

### Mercury dynamics

**Fig 8:**
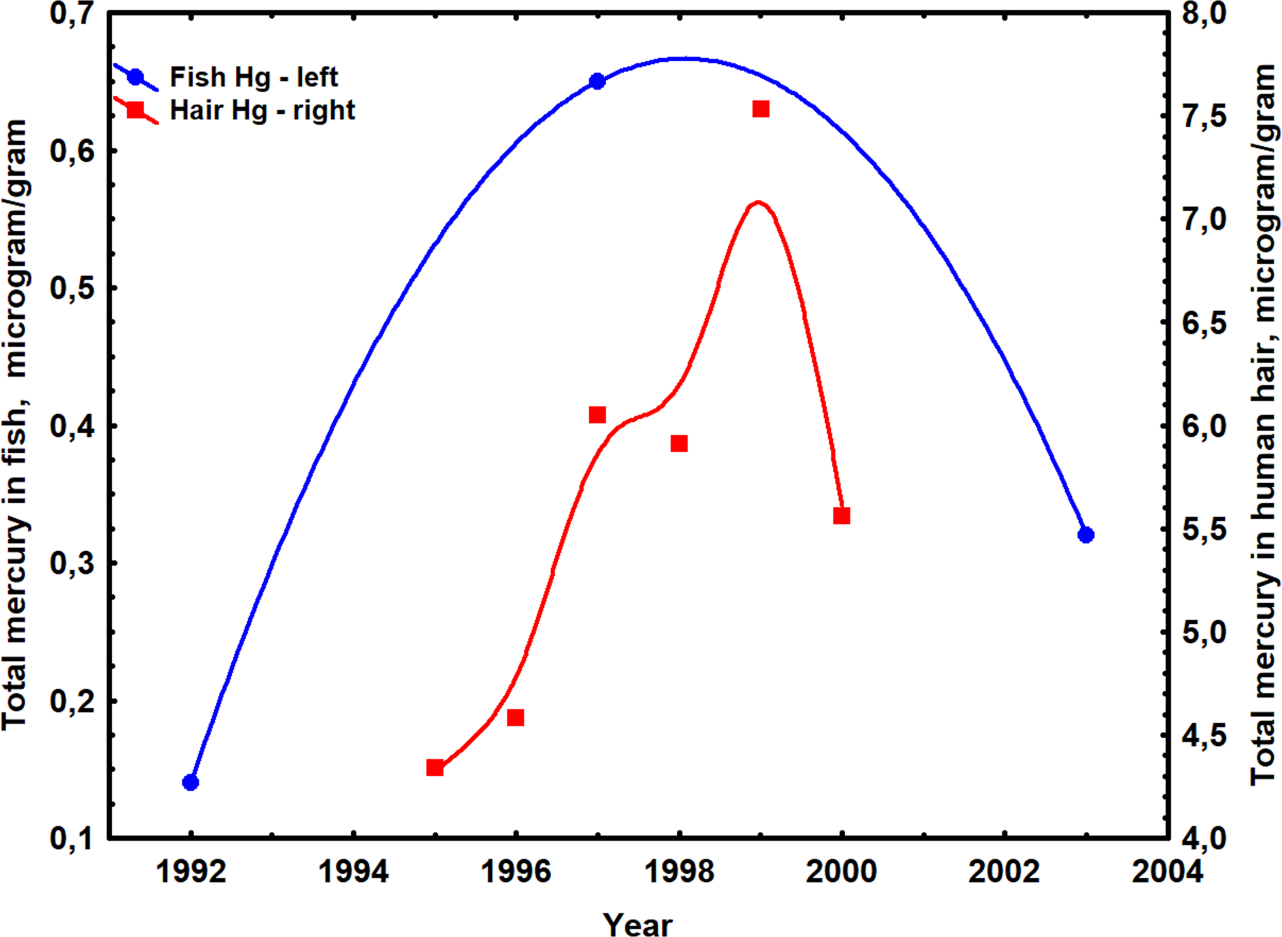
Historical variation of mercury levels in *Cichla spp*.(blue line) and in the hair of fish-eating residents (red line) from Balbina Reservoir following impoundment. Data from [95] and B. Forsberg, unpublished.

Average size normalized mercury levels in *Cichla* spp. from Balbina Reservoir increased from a low of 0.15 μg g^-1^ in 1992 to a peak of 0.65 ppm in 1997, 10–11 years after impoundment, and then declined to 0.32 μg g^-1^ by 2003 (Fig 8). Average mercury levels in the hair of local human populations who consumed these fish as their main protein source followed a similar trend (Fig 8) [95], rising from an initial value of about 4.3 μg g^-1^ in 1995 to a peak of 7.5 μg g^-1^ in 1999 and then declining to 5.6 μg g^-1^ by 2000.

## Discussion

### Impact on the downstream sediment supply

Theoretical and historical evidence from other impounded rivers indicates that the 900 x 10^6^ tons y^-1^ decrease in sediment discharge expected if the six new Andean dams are built will have serious consequences for fluvial and riparian ecosystems downstream from these sites [98]. The nature and timing of these impacts and the extent to which they propagate through the fluvial ecosystems will depend on the hydrologic and geomorphic characteristics of the river system, which vary from the Andean forelands to the Amazon Delta (Fig 1). Based on results from the Amazon [20] and other river systems [11,14], the largest impacts are likely to occur in the foreland reaches closest to the dams, where large reductions in sediment supply and moderate reductions in peak water discharge are expected to result in erosion and incision of channel beds and decreased rates of channel migration. The gradual coarsening or stripping of finer sediments is expected to produce armored, rocky beds in the cobblestone/sand reaches closest to the dams and coarse sand beds throughout most of the foreland basin reaches.

Williams and Wolman [11] evaluated increases in channel-bed depth (incision) downstream from 6 storage reservoirs along the Colorado and Missouri rivers, comparable in size to those planned for the Grande and Inambari Rivers. The authors analyzed post-impoundment variations in channel morphometry and developed empirical models to predict the maximum increase in channel depth at each cross-section, d_max_ (m), as well as the time it would take to reach 50% and 95% of this maximum value, T_0.5_ and T_0.95_, respectively. Average d_max_ values below these dams ranged from 1.4–4.9 m with T_0.5_ values ranging from 4–13 years. The remaining change was slower due to an increase in particle sizes and the development of armored beds with average T_0.95_ values from 83–246 years. The length of these degraded reaches and the rate at which they expanded downstream varied between sites, depending on inputs of sediments from tributaries downstream from the dams [14]. After twenty years they extended from 15 to greater than 120 km.

The extent of channel incision below larger dams, like those planned for the Ucayali, Marañón and Huallaga rivers, is expected to differ is some respects. Bed elevations in the Nile River below the Aswan High Dam were found to decrease 0.3–0.7 m after impoundment [99] but it was not known how far downstream these changes extended. Dai and Lui [10] found much larger changes in the channel of the Yangtze River downstream from the Three Gorges Dam with evidence of incision depths ranging from 1–2.5 meters and extending 670 km below the impoundment. The degree of incision below larger dams thus appears to be similar to that below the smaller dams but can extend much farther downstream.

As bed elevations in foreland rivers decline, river surface levels are expected to fall, reducing inputs of water, sediments and nutrients to adjacent floodplain environments during seasonal floods. In addition to reducing floodplain fertility, this is expected to alter spatial patterns of inundation, critical to floodplain plants [21, 35]. Reduced connectivity between channels and floodplains could also affect the mobility of human populations and the movements of fish and other aquatic organisms that migrate between these environments to feed or reproduce.

Analyzing the temporal dynamics of river channels and floodplain geomorphology across the Amazon basin, Constantine *et al*. [20] demonstrated that the rate of channel change, the rate of channel avulsion and the frequency of oxbow lakes were all positively correlated to width normalized sediment discharge and that the largest effects occurred in the Amazon foreland regions. Based on their findings, we expect the massive reductions in sediment discharge predicted for the foreland reaches below the Andean dams to result in a major reduction in the rates of channel migration and avulsion and in the frequency of oxbow lakes along these channels. Based on studies of forest dynamics in this same region [100], we expect the decline in channel migration to result in a general decrease in plant diversity in both terrestrial and aquatic environments. Increased stability and reduced inundation of the floodplains along these reaches is also likely to promote agricultural development with accompanying increases in deforestation. Rates of erosion and deposition in both channels and alluvial wetlands are also expected to decline [20], reducing the fertility and sediment storage capacity of these environments.

The Sub-Andean forelands have trapped about 50% of all the sediment leaving the Andes throughout the last ~10 M years and continue to do so to varying degrees in the era of instrumental sediment measurements [63, 61]. In the Beni River, for example, almost half of the 243 x 10^6^ tons y^-1^ of sediment passing by the Angosto del Bala gorge is currently deposited in the subsiding foreland basin [18, 98]. Similar levels of storage have been reported in the forelands of the Grande and Marañón Rivers [18, 63]. A decrease in the rates of sedimentation in these foreland basins could therefore have a significant effect on the sediment balance of these regions.

The extent to which reduced sediment loads and their effects propagate through the lowland river system will depend on the magnitude of sediment inputs from downstream tributaries and lowland channel-floodplain exchange processes. Results from other large river systems provide some insight into the expected trends. In the Yangtze River below the Three Gorges Dam, which receives sediment inputs from minor tributaries and channel erosion, sediment discharge was 40% lower than pre-impoundment levels 1200 km below the dam [10]. In the Nile River below the Aswan High Dam which has no significant downstream tributaries, sediment loads were still 80% below pre-impoundment levels 965 km downstream from the impoundment [6]. In the Mississippi River, which has dams retaining sediments along all of its major tributaries, the median sediment load just above its delta is currently 73% below pre-impoundment levels [101], despite large tributary inputs. A different pattern might be expected below the Andean dams where most tributaries are still largely unregulated. The foreland and lowland tributaries below the six Andean dam sites drain about 31% of the Andean highlands and presumably account for about 29% of the current sediment load of the Amazon main channel, assuming the Andes yield is 93% of the total [4]. Inputs from rivers draining the Guyana and Brazilian Shields could increase this proportion by up to 7% [4]. Downstream sediment sources can therefore expected to maintain the sediment load of the lower Amazon main channel at most at 36% of its pre-impoundment level.

Channel erosion and deposition have been shown to play a major role in the sediment dynamics of the lowland Amazon. A detailed sediment balance for the 1560 km reach of the Amazon mainstem between São Paulo de Olivença and Obidos [19] (Fig 1) demonstrated that sediment exchanges due to bank erosion (1570 x10^6^ tons y^-1^), bar deposition (380 x10^6^ tons y^-1^) and floodplain sedimentation (1690 x10^6^ tons y^-1^) were of same order of magnitude as the total tributary input (1448 x 10^6^ tons y^-1^). The cumulative result of tributary inputs and channel exchange processes along the entire reach was a net reduction of at least 200 x10^6^ tons y^-1^ in the downstream sediment flux, which was deposited along the central Amazon floodplain. An additional 300–400 x 10^6^ tons y^-1^ are currently deposited on the Delta plain below Obidos [19]. Based on the relationships reported by Constantine *et al*. [20], we expect the magnitude of channel exchange processes to decline as main channel sediment loads decrease following impoundment while the lowland floodplain and delta regions should continue to act as a net sink for sediments in the region.

If the initial drop in sediment discharge caused by the Andean dams is replenished only by downstream tributaries and the forelands and the mainstem lowlands continue acting as net sediment sinks, the sediment load of the Amazon mainstem is expected to fall by at least 64% and this could have major consequences for the central floodplain and delta regions. Channel bed scouring is expected to reduce hydrological connectivity between channels and floodplains and alter the inundation regimes in floodplain lakes and wetlands, with associated impacts on aquatic flora and fauna [21, 35, 37]. The reduction of sediment concentrations will lead to a large reduction in the quantities of suspended sediment decanted into the floodplain via floodplain channels and diffuse overbank flow [19]. The mainstem lowlands are the most densely populated regions in the Amazon and the most important for agriculture and fish production. Decreased inputs of these nutrient rich sediments could alter the morphology and hydrology of these depositional environments [9, 20] and potentially reduce their agricultural and aquatic productivity.

### Impact on the downstream nutrient supply

While the expected decrease in basin-wide nutrient supplies due to the dams was somewhat lower than that predicted for sediments, especially for N, the predicted changes are considerable. Since these reductions are associated predominantly with fine sediments, they are expected to extend to the lowland floodplains and delta and impact the nutrient balance in these regions. We estimated that supplies of both PP and PN to these depositional environments will decline by 62%. The impact of this decrease on soil fertility and agricultural production in these regions is unclear. Based on mineralogical analyses, Martinelli *et al*. [23] concluded that soils along the central Amazon floodplain were derived predominantly from Andean rocks. Chemical analyses showed them to be fertile, with elevated levels of Na, Mg, K, Ca, P and ion exchange capacity [23, 24, 25], especially when compared to ancient *terra firme* and black water floodplain soils. These nutrients are leached slowly from the mineral component of the soil and will presumably be released for many years without additional alluvial inputs. In contrast, the levels of N in central Amazon floodplain soils are low, relative to the nutrient requirements of floodplain forests, and may require regular inputs from the river and other sources to maintain soil fertility and productivity [24]. In an analysis of the nitrogen balance of a central Amazon floodplain lake, that included extensive areas of seasonally inundated agricultural soils, Kern and Darwich [102] found that net inflow from the river accounted for 50% of the total input of N to the lake with the remainder coming from atmospheric deposition and nitrogen fixation. These inputs were offset by losses from denitrification, resulting in only a small net annual gain in system fertility. Considering the dynamics of N in this system and its apparent deficiency relative to other plant nutrients, a large reduction in the supply of PN to lowland floodplains is likely to result in a decline in soil nitrogen and decreased aquatic agricultural productivity in these regions.

A reduction in the nutrient supply to mainstem floodplains is also expected to lower TP concentrations in Amazon floodplain lakes. TP concentrations in these lakes depend, in part, on nutrient inputs from associated rivers [102]. Andean tributaries flowing through the central Amazon lowlands are high in TP [5, 103] and the TP levels in their associated floodplain lakes are about twice as high as those associated with nutrient-poor clear and black water rivers [26, 76]. There is evidence that only part of the P associated with river particulates is bioavailable [104]. However correlations encountered between the concentration of TP and both the biomass and production of phytoplankton in central Amazon floodplain lakes [26, 27, 76], suggest that lower inputs of riverine particulates after impoundment could also reduce phytoplankton production. This, in turn, could result in a reduction in commercial fish yield, 40% of which is derived from phytoplankton based food webs [105, 106]. Goulding *et al*. [16] attributed the collapse of the planktivorous mapará industry in lower Tocantins River to a reduction in phytoplankton biomass following the construction of the Tucurui dam. Merona *et al*. [107] attributed the general decline in aquatic productivity and fish catch per unit effort below Tucurui dam to the reduction in nutrient levels. Historic declines in fish yield in the Nile River and coastal areas downstream from the Aswan High Dam have also been attributed to lower levels of nutrients and aquatic primary production associated with nutrient retention by the reservoir [7, 8].

The reduction in the N:P ratio of the downstream nutrient supply, due to the selective retention of P-rich sediments following impoundment, could also affect the patterns of nutrient limitation in lowland floodplain plants, intensifying the tendency toward N-limitation already identified in these environments [24, 26, 27, 76, 102, 108].

### Impact on the river flood pulse

The observed decreases in the range of mean monthly stage heights of the Uatumã river below Balbina Dam (Fig 4A) and the Tocantins River below Tucurui Dam (Fig 4C) are typical of most hydroelectric storage dams where stage and discharge fluctuations are reduced to stabilize power generation [11, 42, 43, 44]. Near permanent flooding in the low lying parts of the floodplains below these dams, due to increased stage heights at low water, was expected to impact the alluvial vegetation occupying these regions. A recent study of alluvial forests below Balbina Dam demonstrated that all trees occupying low-lying areas of the floodplain are now predominantly dead [109].Tree ring analyses and C14 dating of these dead trees indicated that most of them died in the period 2001–2010 when the lower floodplain was permanently inundated for multiple years [109]. Trees occupying the highest elevations on the Uatumã floodplain, whose phenologies are normally linked to seasonal flooding cycles [37, 38], would have also been impacted since these areas are now almost permanently dry (Fig 4A). The large reduction in peak flooded area expected on the floodplain below Balbina Dam (~37%) could have also reduced fish yields in this region [39–41].

Changes in flood period were observed at nearly all elevations on the Uatumã floodplain (Fig 4B) and at all but the highest elevations on the Tocantins floodplain (Fig 4D). The distribution, biomass and diversity of plants along Amazonian floodplains have been shown to be closely correlated to flood period, with the highest community biomass and diversity being associated with the shortest flood periods [31, 34, 35, 38, 45]. Seasonal cycles of growth, leaf fall, seed production and dormancy in these species are all synchronized with the annual flood cycle [37, 38]. The disruption of these patterns due to changes in the spatial distribution of inundation periods would affect the entire floodplain plant community and also the fish, bird, primate and insect communities which depend on these plants for their own growth and reproduction, as demonstrated in similar environments [15, 32, 33, 36].

The impact of impoundment on the flood pulses below the six Andean storage dams will depend on the discharge management regimes employed by their operators and the geomorphological characteristics of the river channels and floodplains present in the downstream reaches. The changes in stage height variation observed below Balbina and Tucurui reflect the DMRs employed at these dams and the observed differences between dams indicate the range of changes expected below the Andean dams. The impact of these changes on floodplain environments below the Andean dams will depend on the topography of these areas relative to river level fluctuations [21]. The rivers below Balbina and Tucurui dams are both low gradient rivers with relatively low concentrations of suspended sediment [107, 109]. Due to low sediment inputs, floodplain topography is variable without levees at the river margins. Floodplain environments are well connected to the river and are only isolated completely at extreme low water. As a result, changes in flood periods on the floodplain, following impoundment, were directly correlated to variations in river stage height (Fig 4). The floodplains downstream from the Andean dam sites are all associated with medium-high gradient rivers with high concentrations of suspended sediments [18, 61]. Due to large sediment inputs during seasonal floods, these floodplains tend to be elevated and uniform in topography and are covered primarily with alluvial forest, oxbow and scroll bar lakes [29, 100]. Due to the high gradient and sediment load of the rivers, they are subject to rapid lateral erosion and channel change [20] which results in unstable levees penetrated by numerous scroll bar channels [29, 100]. These channels allow exchange of water between river and floodplain, resulting in a high level of connectivity throughout much of the hydrological cycle [29]. As with Balbina and Tucurui, then, we expect a close correlation between changes in river stage variability and flood periods on these floodplains. However, since higher elevations tend to predominate on the Andean floodplains, a reduction in mean stage height at high water could result in a much larger portion of the floodplain being permanently isolated than was observed below Balbina and Tururui dams. This could have profound impacts on the biota occupying these areas, especially on the phenology of floodplain plants [37, 38] and the survival of animals that depend on these plants [15, 32, 33]. The isolation of large parts of the floodplain may also promote human occupation in these areas and accelerate rates of deforestation in alluvial forests.

### Impact on downstream fish production

The strong positive correlation found in the Loreto Region between annual fish yield and peak flooded area two years before capture (Table 4, Fig 5) is consistent with the observation that the Loreto fish yield was composed predominantly of short lived species. *P*. *nigricans*, the most important species in the Loreto fishery [74, 75], has a fisheries recruitment age of approximately 1.5 years [110, 111]. A similar time lag (~2 years) between peak flooding and fish yield was observed by Wellcome [39] in several African river systems, by Merona and Gascuel [40] in an Amazon floodplain lake and by Isaac *et al*. [41] for multispecies fisheries in the lower Amazon, but not by Castello *et al*. [73] for detritivorous fish yields in this same region. Wellcome attributed the time lag in the African relationships to the positive effects of increased flooding on the growth and survival of young of the year (YOY) which are captured as adults 1–2 years later. The survival of YOY of *P*. *nigricans* and other short-lived species in the Loreto Region depends on the availability of flooded forests and other vegetated habitats which provide a refuge from larger predators. During large floods, the size of this refuge and the probability of survival would have increased considerably, contributing to greater fish yields when these YOY were captured 1.5–2 years later.

If a reduction in peak stage height and flooded area like that observed below Balbina dam were to occur below the TAM 40, Pongo de Aguirre and Pongo de Manseriche dams, the effect on fish yield in the Loreta Region would be dramatic. Based on the predictive model presented here (Fig 6), a 37% reduction in maximum flooded area in the Loreto Region would result in an 88% decline in annual fish yield. Since a similar change in peak stage height was not observed below Tucurui Dam, this should be considered the worst case scenario. The 3 Andean dams planned for the Madeira river system are also expected to reduce peak flooded area and fish yields in the extensive fluvial wetlands downstream from these sites. An additional decrease in fish yield is expected due to the decline in nutrient supply to these rivers and floodplains following impoundment.

### Impact on upstream fish production

The areal fish yield estimated for the Andean reservoirs (2.1 tons km^-2^ y^-1^) was similar to the maximum value registered in Tucurui Reservoir after impoundment, 1.7 tons km^-2^ y^-1^ [107]. Areal fish yields in the Andean dams are expected to be more stable than those encountered in lowland Amazon dams, since the nutrients that sustain them will come predominantly from inflowing Andean tributaries and not from decaying terrestrial vegetation [54, 107]. Total fish yields estimated for the Andean reservoirs (Table 5) could compensate, in part, for the loss in fish yields expected below the dams (Fig 6). The estimated fish yield in Manseriche reservoir (15,200 tons km^-2^ y^-1^), for example, is similar to the average fish yield of the Loreto Region (19,400 tons km^-2^ y^-1^). However, the impact on the livelihoods, economy and protein supply of the populations downstream from the dams will likely be severe, and mercury contamination both above and below the dams could create additional issues (see below).

### Reservoir sedimentation

Besides the impacts on downstream sediment and nutrient supplies, reservoir sedimentation can also affect dam operation, the quality of benthic habitats and the pattern of human occupation and activities in the reservoirs. Dam operation will be affected if a reservoir fills to the point that sediment begins to enter the turbines. While this can be mitigated or delayed by dredging and flow management, when sediment levels reach the turbine inflow it often marks the end of the useful life of the power plant. If and when this occurs depends on the rate of filling (Table 6), the spatial distribution of sedimentation and the depth of the turbine inflow. For the Manseriche and Inambari reservoirs where complete fill times were estimated at 6240 and 1225 years, respectively, sediment damage is unlikely to occur before other factors terminate dam operations [112]. In contrast, the complete fill times for Angosta del Bala and Rositas reservoirs, 106 and 126 years, respectively, are near the expected useful lives of most dams (50–100 years [112]), and fill times to the turbine inflow are expected to be considerably shorter, which could affect the technical viability of these dams.

Several environmental impacts linked to sedimentation must also be considered. Deposits of fine riverine particulates will cover the coarse gravel substrates originally encountered on the river bed, impacting the benthic fauna and fish which use this habitat for feeding and spawning. Bottom sediments will accumulate first near the inflowing tributaries, where new wetlands are expected to develop. These wetlands could provide new habitats for regional flora and fauna, but could also provide new sources of greenhouse gases and sites for mercury methylation. Gold mining of alluvial deposits in the basin upstream from the dam site are currently restricted to narrow floodplain environments, due to the high risk of mining in the fast flowing river channels. The extensive deposits of alluvial sediments expected to accumulate near tributary mouths in the reservoir will provide an ideal environment for placer mining and is likely to lead to an expansion of these activities in the region. The expansion of mining and other activities upstream of the reservoir could increase the flux of sediments and associated mercury to the reservoir, accelerating siltation and mercury contamination.

### Greenhouse gas emissions

The average daily areal emission rate for the 4 reservoirs during the first 30 years of operation, 2,890 mg C-CO_2_e m ^-2^ d ^-1^, was somewhat lower than the average value of 3,817 C-CO_2_e m ^-2^ d ^-1^ reported for Amazon reservoirs [82, supplemental data]). This is not surprising considering the expected change in emissions over time (Fig 7) [47] and that most Amazon reservoirs are much younger than 30 years. However, considering the combined surface area of these reservoirs (8812 km^2^), they could have a significant impact on global emissions. Based on recent review of emissions from reservoir surfaces world-wide [113], the sum of 30 year-average emissions from all four hydroelectric reservoirs (80.9 X 10^5^ tons C-CO_2_e y^-1^, Table 7) would increase global emissions from this source by 1%. Downstream emissions were not considered in this estimate since data for this emission component are lacking for most dams.

An average of 17% of total carbon emissions (16.6 x 10^5^ tons C-CO_2_e y^-1^, Table 7) is expected to occur downstream of the 4 Andean dams. Downstream emissions have been shown to contribute significantly to total emissions in other Amazonian dams [47, 86, 87, 88] and should always be considered in environmental impact assessments of these systems.

Whether the values presented in Table 7 should be considered net or gross emissions will depend on the expected changes in the regional carbon balance following impoundment. Net emission, in this context, represents the net difference between the balance of CO_2_ equivalent carbon in the reservoir landscape before and after impoundment. Most of the area to be inundated by the four Andean reservoirs is currently occupied by tropical broadleaf forest, while a smaller fraction is occupied by river channels and fluvial wetlands. A recent review of C mass balance analyses for the lowland Amazon forest [114] concluded that this system is approximately carbon neutral. Several regional analyses have estimated high gross CO_2_ equivalent carbon emissions from river channels and wetlands in the lowland Amazon [115, 116, 117]. However when carbon uptake by aquatic plants was also considered in more complete mass balance analyses [26, 118] these systems were also found to be approximately carbon neutral. The errors involved in all of these mass balance analyses are too large to determine with confidence whether these systems are carbon neutral, net sinks of net sources of carbon for the atmosphere, and no similar mass balances are available for the forests and wetlands in the Andean region. Based on the results for lowland regions cited above, it is likely that the forests and wetlands currently occupying the areas of the projected Andean reservoirs are also close to carbon neutral and that the emissions released from these areas once inundated (Table 7) will represent net values. We do not consider in this analysis the assimilation of atmospheric CO_2_ by the terrestrial forest during the period its biomass was growing, since this occurred over millennia. CO_2_ equivalent carbon fluxes derived from the decomposition of this biomass once submerged are therefore considered net emissions. We also ignore CO_2_ emissions associated with aquatic primary production in the reservoir, since these are balanced with recent CO_2_ fixation. However methane emissions associated with aquatic production could contribute significantly to net CO_2_ equivalent emissions, due to the difference in global warming potential of the fixed CO_2_ and released CH_4_ [85].

With greenhouse gas emissions rising globally and the consequences of global warming becoming increasingly apparent, the choice of power generating technologies often involves a comparison of Carbon Emission Factors (CEFs). Bosi [119] estimated CEFS for thermoelectric power plants burning natural gas, fuel oil and coal in Brazil to be 0.115, 0.205 and 0.257 tons C MWh ^-1^, respectively. All of the hydroelectric dams considered here had CEFs below these fossil fuel alternatives (Table 7), with the exception of the Manseriche, which had a predicted CEF (0.206 tons C-CO_2_e MWh ^-1^) similar to an oil-fired power plant and almost twice as high as a gas-fired power plant. Considering the abundance of natural gas reserves in the Amazon Region and the large environmental impacts associated with dams, alternative energy sources should be considered in the case of Manseriche Dam.

### Mercury dynamics

The historical pattern of mercury contamination in *Cichla sp*. in Balbina Reservoir (Fig 8) was similar to that encountered for large predatory fish like *Esox sp*. in north temperate reservoirs, with a gradual rise and fall of mercury during the first 10–25 years after impoundment [50, 51, 120]. Peak mercury levels in north temperate storage reservoirs were found to occur 3–13 years after impoundment with mercury concentrations returning to pre-impoundment levels 10–25 years after impoundment [50, 51, 120]. These trends have been attributed to a gradual rise in the methylation and bioaccumulation of inorganic mercury, naturally present in these systems, due to the slow decomposition of inundated terrestrial vegetation that generates anoxic conditions favorable for methylation. As the terrestrial carbon stock is exhausted and oxygen concentrations rise, the levels of methylation and contamination in aquatic biota return to their pre-impoundment values. Mercury levels in *Cichla sp*. rose during the first ten years after impoundment reaching a peak value of 0.65 μg g^-1^ between 1997 and 1998 (Fig 8). The level then declined during the next 6 years and had still not returned to pre-impoundment levels 25 years after impoundment [53]. The peak level of Hg observed in *Cichla sp*. exceeded the value of 0.5 μg g^-1^ recommended by the WHO and the Brazilian federal government for safe consumption.

Amazon reservoirs differ from north temperate impoundments in that they often have commercial fisheries that exploit the large fish populations which commonly develop in these systems. Fishing communities at Balbina and other Amazon reservoirs obtain most of their protein from reservoir fish and are effectively the top predators in the reservoir food chain. It is not surprising then that the historical trend in the levels of mercury in the hair of Balbina fishermen’s wives was similar to those found in fish (Fig 8) with peak mercury levels in hair occurring in 1991, just 1–2 years after the peak in fish values. Mercury concentrations in hair were higher than those in fish reaching a maximum level of 7.5 μg g^-1^. Concentrations exceeded the conservative reference limit of 1 μg g^-1^ established by the USEPA during the entire study period and exceeded the reference limit of 6 μg g^-1^ established by the WHO between 1997 and 2000, indicating an elevated health risk for this population.

Mercury contamination is also expected below hydroelectric reservoirs as anoxic hypoliminetic waters, rich in MeHg, are released through the turbines to the downstream river channel. Kasper *et al*. [53] found elevated MeHg concentrations up to 200 km downstream from Balbina Dam. Fish below Balbina and Samuel dams were shown to have higher levels of Hg contamination than those found in the respective reservoirs [52, 53] due to the contaminating effect of turbine discharge.

Similar patterns are expected in the six Andean reservoirs with some important differences. All of these reservoirs will inundate extensive terrestrial forests which are expected to decompose and create conditions conducive to mercury methylation for at least 10. During this period, mercury levels are expected to increase in reservoir fish and in human populations that consume these fish. However, unlike most reservoirs in the lowland Amazon and north temperate regions, the anoxic conditions in the Andean reservoirs may continue well beyond the point when terrestrial carbon sources are exhausted due to high levels of primary production fueled by Andean nutrients. External nutrient loading is expected to be high and constant in these systems, resulting in eutrophic reservoirs with permanently anoxic hypolimnia. These stable anoxic environments will promote the methylation and bioaccumulation of mercury in aquatic biota and fish-eating human populations upstream and downstream of the dams for the useful life of the reservoir. Inputs of mercury from gold mining activities upstream of these dam could also contribute to higher levels of contamination in these systems. The high sustained fish yields predicted for these reservoirs (Table 5) are expected to attract fishermen interested in exploiting this resource. These people are likely to consume large quantities of reservoir fish and bioaccumulate significant levels of mercury. If the mercury levels in these populations and in riverine communities downstream from the dam reach those encountered in Balbina reservoir (Fig 8), they would represent a serious health risk, especially for children and pregnant women.

## Conclusions

**Fig 9:**
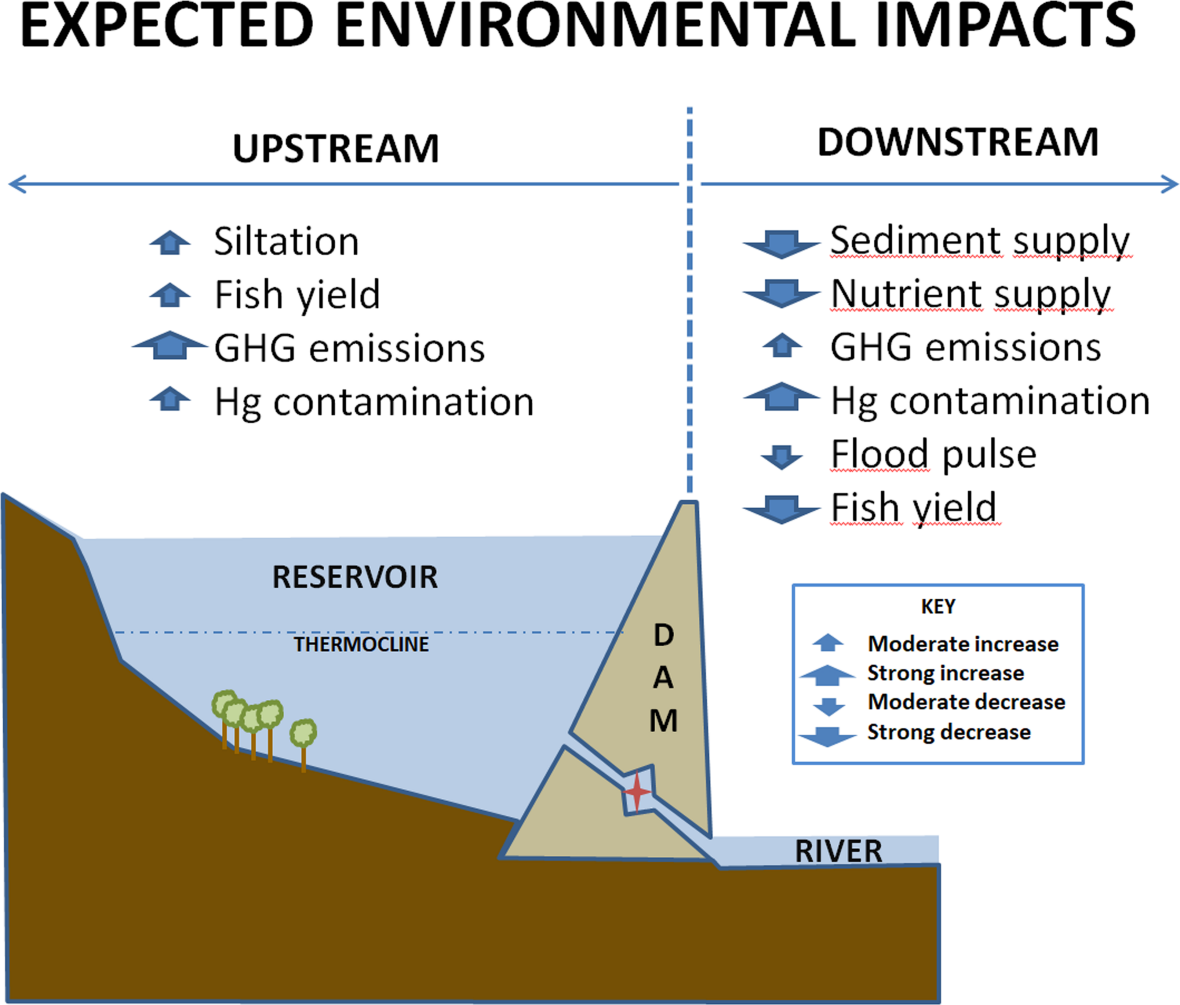
Summary: Expected environmental impacts above and below Andean dams.

The construction of six Andean dams will have extensive impacts on Amazon fluvial ecosystems that need to be addressed in regional infrastructural development plans. Fig 9 summarizes the principal impacts considered here, indicating their relative magnitude and importance to ecosystem structure and functions. Understanding the full complexity of these impacts and their economic costs should inform strategic discussion of whether alternative energy sources could meet growing energy needs while preserving the critical natural resources of the Amazon River basin.

## Supporting information

S1 Fig Relationship between particulate P and total suspended sediments (TSS) for the Amazon mainstem and its principal Brazilian tributaries. Unpublished data from CAMREX Project, http://dx.doi.org/10.3334/ORNLDAAC/904.
(TIF)

S2 Fig Relationship between % N in sediments and total suspended sediments (TSS) in the Amazon mainstem and its principal Brazilian tributaries. Unpublished data from CAMREX Project, http://dx.doi.org/10.3334/ORNLDAAC/904.
(TIF)

S1 Table Historical fisheries and flooding data for the Loreto fishing region of Peru. Flooded areas simulated using the MGB-IPH Large Scale Numerical Hydrological Model, developed specifically for the Amazon Basin [67, 68]. Fisheries data supplied by DIREPRO (Dirección Regional de Producción, Peru). TY = total annual fish yield, tons y^-1^; YFZ = annual fish yield for regularly exploited fishing zones, tons y^-1^; FZ = number of fishing zones. FZ was assumed to represent fishing effort. Mifa = minimum annual flooded area, km^2^; Mxfa = maximum annual flooded area, km^2^.
(XLSX)
