# Supplementary figures and images for: The potential impact of new Andean dams on Amazon fluvial ecosystems
Source: PLoS One. 2017 Aug 23;12(8):e0182254. doi: 10.1371/journal.pone.0182254 (PMC5568116; doi:10.1371/journal.pone.0182254)

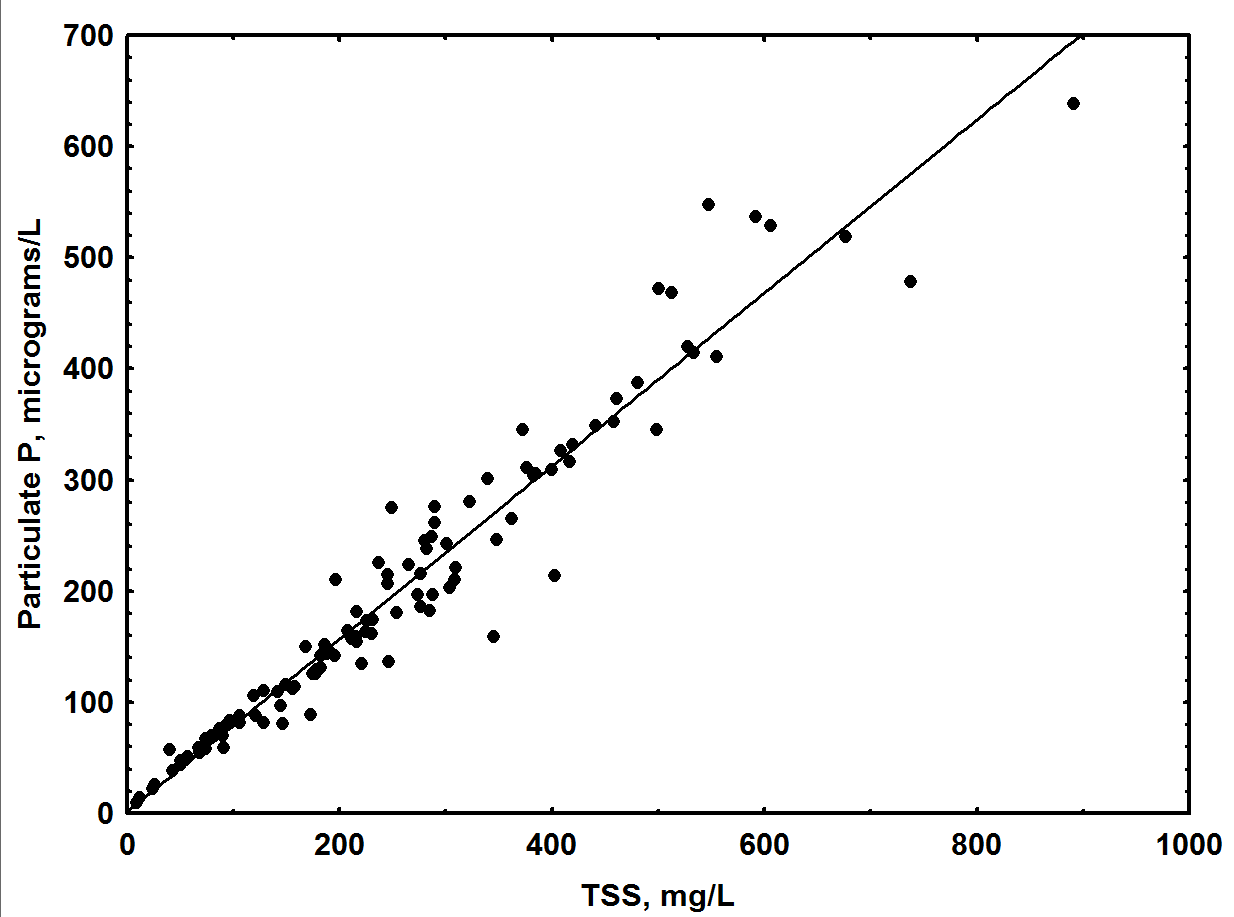

Supplement: S1 Fig — Unpublished data from CAMREX Project, http://dx.doi.org/10.3334/ORNLDAAC/904. (TIF) [file pone.0182254.s001.tif]

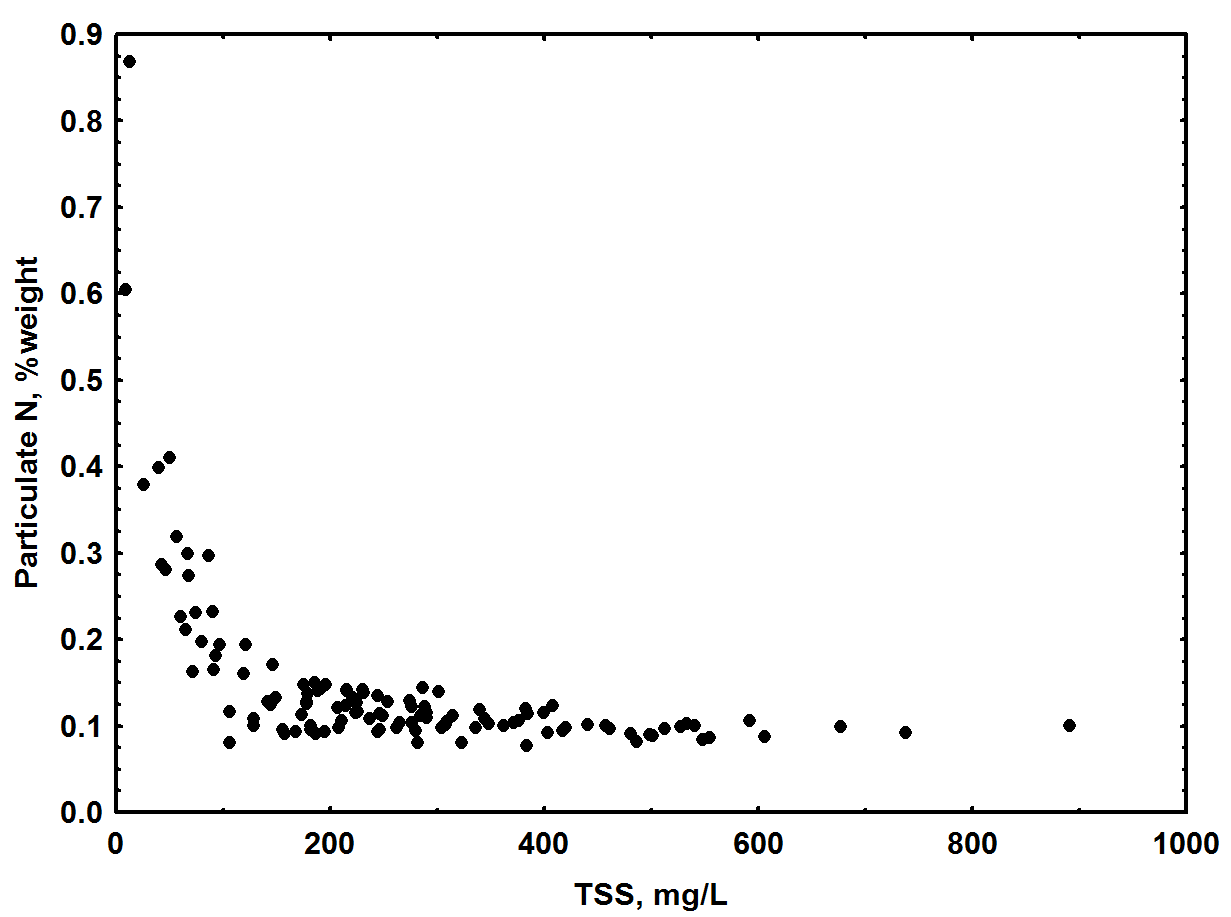

Supplement: S2 Fig — Unpublished data from CAMREX Project, http://dx.doi.org/10.3334/ORNLDAAC/904. (TIF) [file pone.0182254.s002.tif]
